# Exposure to persistent organic pollutants alters the serum metabolome in non-obese diabetic mice

**DOI:** 10.1007/s11306-022-01945-0

**Published:** 2022-11-03

**Authors:** Tim Sinioja, Johanna Bodin, Daniel Duberg, Hubert Dirven, Hanne Friis Berntsen, Karin Zimmer, Unni C. Nygaard, Matej Orešič, Tuulia Hyötyläinen

**Affiliations:** 1 School of Science and Technology, Örebro University, 702 81 Örebro, Sweden; 2 Division of Infection Control and Environmental Health, Norwegian Institute of Public Health, 0456 Oslo, Norway; 3 Norwegian University of Life Sciences, 1432 Ås, Norway; 4 National Institute of Occupational Health, 0363 Oslo, Norway; 5 School of Medical Sciences, Örebro University, 702 81 Örebro, Sweden; 6 Turku Bioscience Centre, University of Turku and Åbo Akademi University, 20520 Turku, Finland

**Keywords:** Exposomics, Metabolomics, Persistent organic pollutants, Perfluorinated alkyl substances, Environmental exposure, Type 1 diabetes

## Abstract

**Introduction:**

Autoimmune disorders such as type 1 diabetes (T1D) are believed to be caused by the interplay between several genetic and environmental factors. Elucidation of the role of environmental factors in metabolic and immune dysfunction leading to autoimmune disease is not yet well characterized.

**Objectives:**

Here we investigated the impact of exposure to a mixture of persistent organic pollutants (POPs) on the metabolome in non-obese diabetic (NOD) mice, an experimental model of T1D. The mixture contained organochlorides, organobromides, and per- and polyfluoroalkyl substances (PFAS).

**Methods:**

Analysis of molecular lipids (lipidomics) and bile acids in serum samples was performed by UPLC-Q-TOF/MS, while polar metabolites were analyzed by GC-Q-TOF/MS.

**Results:**

Experimental exposure to the POP mixture in these mice led to several metabolic changes, which were similar to those previously reported as associated with PFAS exposure, as well as risk of T1D in human studies. This included an increase in the levels of sugar derivatives, triacylglycerols and lithocholic acid, and a decrease in long chain fatty acids and several lipid classes, including phosphatidylcholines, lysophosphatidylcholines and sphingomyelins.

**Conclusion:**

Taken together, our study demonstrates that exposure to POPs results in an altered metabolic signature previously associated with autoimmunity.

**Supplementary Information:**

The online version contains supplementary material available at 10.1007/s11306-022-01945-0.

## Introduction

Type 1 diabetes (T1D) is one of the most common chronic metabolic disorders in children and adolescents, with an estimated worldwide incidence of 128,900 new cases and corresponding prevalence of nearly 1,100,000 existing cases in the under 20 year age group (Patterson et al., 2019). Genetic factors are known to play a significant role in the development of autoimmune disorders, including T1D in humans, potentially in combination with environmental factors (Bach, 2001; Regnell & Lernmark, 2013; Rook, 2012). In recent decades, age at diagnosis of T1D has declined (Group, 2008; Harjutsalo et al., 2013), suggesting that the impact of environmental factors during early development has increased. Factors such as maternal infections or infections in early life, deficiency of specific nutrients during pregnancy and/or early childhood, have been associated with risk of T1D in observational studies (Stene & Gale, 2013). Other proposed risk factors of T1D include alterations in gut microbiota (Chatenoud et al., 2012) and exposure to environmental chemicals during development (Bodin et al., 2015). However, the impact of environmental factors on disease development is not well characterized. While epidemiological studies have identified specific associations between environmental factors and disease risk, causal relationships require also controlled, experimental in vitro and in vivo exposure models.

Environmental chemicals, particularly persistent organic pollutants (POPs) including per- and polyfluoroalkyl substances (PFAS), organochlorine pesticides (OCPs), polychlorinated biphenyls (PCBs) and brominated flame retardants (BFRs), such as hexabromocyclododecane (HBCD) and polybrominated diphenyl ethers (PBDEs), are commonly detected in humans as demonstrated by the European Human BioMonitoring data dashboard (HBM4EU, 2020). Although humans are exposed to complex, man-made chemical mixtures via several sources and routes, knowledge of adverse effects caused by such chemicals is mainly based on studies focusing on a single or few chemicals at a time. However, combined exposure to multiple chemicals may cause synergistic or additive adverse health effects, even if the levels of all substances included in the chemical exposure mixture are below their individual safety thresholds (JRC, 2018).

Humans are exposed to PFAS, PCBs and BFRs through food (Harrad et al., 2004; Haug et al., 2011; Hites et al., 2004; Karrman et al., 2006), via inhalation (Casey et al., 1999; Mandalakis et al., 2008) and indoor dust (Hazrati & Harrad, 2006; Hwang et al., 2008; Wilford et al., 2004), as well as through breastfeeding (Karrman et al., 2007). After intake and absorption, perfluorinated compounds predominantly associate with proteins and occur at the highest concentrations in blood, liver and kidneys (Karrman et al., 2006; Lau, 2012), while the main site of PCB and BFR accumulation is adipose tissue (Mullerova & Kopecky, 2007). During the early development in humans, POPs can pass the placental barrier, reach and deposit in fetal tissues and organs (Li et al., 2020a; Porpora et al., 2013). They are, in general, found in fetal organs at concentrations lower than in maternal serum, but similar to placental levels (Mamsen et al., 2019).

In living organisms, many POPs are known to cause adverse health impacts. For example, PFAS exposure has been associated with endocrine disruption (Lopez-Espinosa et al., 2012), increased cholesterol levels (Li et al., 2020b), altered metabolic processes in fat and liver (Bassler et al., 2019), and has been linked with autoimmune disorders such as celiac disease (Sinisalu et al., 2020) and type 1 diabetes (McGlinchey et al., 2020). Several PCB congeners have been associated with adverse developmental (Mol et al., 2002), endocrine (Cao et al., 2008; Chen et al., 2008; Vasiliu et al., 2006) and immunological (Heilmann et al., 2006; Lee et al., 2007; Park et al., 2008) effects. Adverse health consequences of exposure to BFRs have also been observed, including thyroid disorders (Stapleton et al., 2011; Zota et al., 2011), diabetes (Lee et al., 2010) and neurodevelopmental (Gascon et al., 2011) health effects.

The number of exposure studies investigating autoimmune disease progression in animal models are limited. We have previously reported that exposure to a single PFAS, perfluoroundecanoic acid (PFUnDA), caused a non-monotonic dose–response effect on the immune system in non-obese diabetic (NOD) mice, where low and intermediate exposures resulted in a delay of onset of autoimmune diabetes, while exposure to very high levels accelerated insulitis development (Bodin et al., 2016). Further, exposure of the NOD mice to a POP mixture containing 7 PCBs, 9 OCPs, 7 BFRs and 6 PFAS (Berntsen et al., 2017) resulted in a trend of decreased peritoneal macrophage phagocytosis (Berntsen et al., 2018). Additionally, an early event of insulitis development, namely a reduced number of F4/80-tissue resident macrophages, was also apparent in the histological analysis of pancreas in these mice. We have previously shown that immunotoxic effects of a POP mixture in in vitro studies of both rodent and human blood-derived macrophages were mainly caused by the PFAS in the mixture (Berntsen et al., 2018).

Human studies investigating early life exposure to POPs and their role in T1D development show inconsistent results. A cross-sectional study including 820 patients with T1D showed a significant association between high PFAS levels in blood serum and lower risk of T1D development in children and adults (Conway et al., 2016). Conversely, elevated perfluorooctanesulfonic acid (PFOS) levels were reported in young patients newly diagnosed with T1D (Predieri et al., 2015), while another birth-cohort study found no evidence that fetal and early life exposure to POP (including 14 PFAS) was a significant risk factor for later T1D development (Salo et al., 2019). Other studies suggest that PFAS exposure is associated with hyperglycemia, serum high-density lipoprotein (HDL) cholesterol and increased blood insulin (Lin et al., 2009), may lead to immunotoxicity (Borg et al., 2013), and modulates neonatal serum phospholipids associated with increased risk of T1D (McGlinchey et al., 2020). Overall, the molecular mechanisms underlying the effects of exposure are still not well characterized.

Herein we investigated metabolic alterations in NOD mice following pre- and postnatal exposure to a mixture of persistent organic pollutants, in which the concentrations of POPs were based on estimated daily intake levels reported in the Nordic population (Berntsen et al., 2017). Since PFAS was the most potent chemical group in the POP mixture regarding macrophage phagocytosis (Berntsen et al., 2018), we focused on possible effects of PFAS on the metabolome.

## Materials and methods

### Exposure mixture

All 29 compounds in the POP mixture (Table 1) were of HPLC/GC–MS grade (at least > 98.5% purity) and underwent a quality check for the absence of any dioxin-like compounds by the suppliers (Chiron AS, Norway; Sigma-Aldrich, Germany; Santa Cruz Biotechnology, Inc., USA).

**Table 1:** Daily exposure doses of POPs via feed in low and high exposure NOD mouse groups

Compound	Low exposure µg/day	High exposure µg/day
Chlorinated compounds		
PCB 28 (2,4,4ʹ-trichlorobiphenyl)	0.018	0.350
PCB 52 (2,2ʹ,5,5ʹ-tetrachlorobiphenyl)	0.041	0.825
PCB 101 (2,2ʹ,4,5,5ʹ-pentachlorobiphenyl)	0.070	1.400
PCB 118 (2,3ʹ,4,4ʹ,5-pentachlorobiphenyl)	0.121	2.425
PCB 138 (2,2ʹ,3,4,4ʹ,5ʹ-hexachlorobiphenyl)	0.173	3.450
PCB 153 (2,2ʹ,4,4ʹ,5,5ʹ-hexachlorobiphenyl)	0.173	3.450
PCB 180 (2,2ʹ,3,4,4ʹ,5,5ʹ-heptachlorobiphenyl)	0.046	0.925
*p,p’*-DDE (4,4ʹ-dichlorodiphenyldichloroethylene)	0.359	7.175
HCB (hexachlorobenzene)	0.150	3.000
α-Chlordane	0.113	2.250
Oxychlordane	0.038	0.750
*trans*-Nonachlor	0.038	0.750
α-HCH (α-hexachlorocyclohexane)	0.065	1.300
β-HCH (β- hexachlorocyclohexane)	0.053	1.050
γ-HCH (γ-hexachlorocyclohexane/lindane)	0.071	1.425
Dieldrin	0.225	4.500
Brominated compounds		
PBDE 47 (2,2ʹ,4,4ʹ-tetrabromodiphenyl ether)	0.121	2.425
PBDE 99 (2,2ʹ,4,4ʹ,5-pentabromodiphenyl ether)	0.024	0.475
PBDE 100 (2,2ʹ,4,4ʹ,6-pentabromodiphenyl ether)	0.019	0.375
PBDE 153 (2,2ʹ,4,4ʹ,5,5ʹ-hexabromodiphenyl ether)	0.004	0.075
PBDE 154 (2,2ʹ,4,4ʹ,5,6ʹ-hexabromodiphenyl ether)	0.008	0.150
PBDE 209 (2,2ʹ,3,3ʹ,4,4ʹ,5,5ʹ,6,6ʹ-decabromodiphenyl ether)	0.188	3.750
HBCD (1,2,5,6,9,10-hexabromocyclododecane)	0.038	0.750
Perfluorinated compounds		
PFHxS (perfluorohexanesulfonic acid)	0.002	0.043
PFOS (perfluorooctanesulfonic acid)	0.033	0.650
PFOA (perfluorooctanoic acid)	0.055	1.100
PFNA (perfluorononanoic acid)	0.018	0.350
PFDA (perfluorodecanoic acid)	0.024	0.475
PFUnDA (perfluoroundecanoic acid)	0.012	0.240

Three exposure diets control (solvents only), low exposure (5000 × human estimated daily intake (EDI)) and high exposure (100,000 × human EDI), were prepared by dissolution of POPs in corn oil intended for human consumption (Yonca Gida Sanayi A.S., Turkey), which was then incorporated into pelleted feed (TestDiet, USA). The EDI values in low and high exposure diets (ng/kg BW/day in humans) were adjusted to a mean mouse bodyweight of 25 g, assuming 3 g per day feed intake, as earlier described (Berntsen et al., 2017). The total average daily intake of OCPs and PCBs was 1.8 µg and 35.0 µg; of BFRs 0.40 µg and 8.0 µg; and of PFAS 0.14 µg and 2.9 µg in the low and high exposure groups, respectively.

### NOD mice study

All animal experiments were approved by the Norwegian Animal Research Authority and conducted in accordance with the Norwegian laws and regulations for experiments using live animals. The study design was reported previously (Berntsen et al., 2018).

Twenty female and ten male NOD/SHiLtJ mice (Jackson Laboratory, USA) were randomized into exposure groups and used for breeding at 8 and 10 weeks of age. The POP exposure via feed started at mating and continued through gestation and early life period until 12 weeks of age. Due to developing a more invasive and destructive insulitis (Bao et al., 2002; Kelemen, 2004), only female offspring were selected at weaning. At 12 weeks of age, eight female offspring were sacrificed in the control group along with seven female offspring from each of the low and high exposure groups.

Blood serum samples were collected and stored at -80 °C until chemical analyses. Samples were prepared and analyzed by mass spectrometry in randomized order.

### Lipidomic analysis

A previously published and modified Folch procedure (Nygren et al., 2011) was used to extract blood serum samples. Briefly, 10 µL blood serum sample aliquots were extracted with 10 µL 0.9% NaCl and 120 µL 2:1 v/v trichloromethane:methanol (Fisher Scientific, UK) solution containing 2.5 µg/mL internal standards purchased from Avanti Polar Lipids Inc., USA and Larodan AB, Sweden (Supplementary Table A1). Samples were vortexed and kept on ice for 30 min prior to 3 min of centrifugation at 9400 × g. Aliquots of 60 µL bottom layer were transferred to new vials and 60 µL of 2:1 v/v trichloromethane:methanol solution were added to each sample.

Calibration standards at concentration levels of 100, 500, 1000, 1500, 2000 and 2500 ng/mL were prepared in 2:1 v/v trichloromethane:methanol solution using supplied by Avanti Polar Lipids Inc., USA and Larodan AB, Sweden chemicals (Supplementary Table A1).

After the extraction, samples were analyzed utilizing ultra-high performance liquid chromatography quadrupole time-of-flight mass spectrometry (UPLC-Q-TOF/MS). An Agilent Technologies 1290 Infinity II system was used, connected to a 6545 Q-TOF/MS with MassHunter B.06.01 software installed to acquire the data. A multisampler, maintained at 10 °C, a column thermostat at 50 °C and a quaternary solvent manager were used to introduce samples onto 1.7 µm, 2.1 mm × 100 mm Acquity UPLC BEH C18 column (Waters Corporation, USA). Solvent and extraction blanks, pure lipid standard samples representing each class of lipids, pooled serum samples and reference plasma samples SRM1950 (NIST, USA) were used to perform a quality control of the analysis. Relative standard deviations (RSD) of peak areas were within accepted analytical limits and were on average 11.9% in lipid standards and 7.3% in pooled control samples.

After the acquisition, the data processing was performed in MZmine 2.18 (Pluskal et al., 2010) applying following steps and options: (1) crop filtering: m/z 350–1200 and retention time (RT) 2.0–12 min; (2) mass detection: noise level at 750; (3) chromatogram builder: minimum time span – 0.8 min, minimum height 1000, m/z tolerance 0.006 m/z or 10.0 ppm; (4) chromatogram deconvolution utilizing the local minimum search algorithm: chromatographic threshold 70%, minimum RT range 0.05 min, minimum relative height 5%, minimum absolute height – 1200, minimum top-to-edge ratio 1.2, peak duration range 0.08–5.0; (5) isotopic peak grouper with the most intense peak as the representative isotope: m/z tolerance 5.0 ppm, RT tolerance 0.05 min, maximum charge 2; (6) peak filter: minimum data points 12, full width at half maximum (FWHM) 0.0–0.2, tailing factor 0.45–2.22, asymmetry factor 0.40–2.5; (7) join aligner: m/z tolerance 0.009 m/z or 10 ppm with weight of 2, RT tolerance 0.1 min with weight of 1, with no requirement of charge state or ID, no comparison of isotope pattern; (8) peak list row filter: feature present minimum In 10% of samples; (9) gap filling using the “same RT and m/z range” algorithm: m/z tolerance 0.009 m/z or 11 ppm; (10) lipids identification using custom database search: m/z tolerance 0.009 m/z or 10.0 ppm and RT tolerance 0.1 min; (11) peak area normalization based on: internal standards of the same lipid classes for identified lipids (PE(17:0/17:0), SM(d18:1/17:0), Cer(d18:1/17:0), LPC(17:0), TG(17:0/17:0/17:0) and PC(16:0/d30/18:1)) and internal standards with closest RT for the unidentified lipids; (12) calculations of concentrations using previously-described 6-point calibration curves. Furthermore, the final dataset was subjected to further data clean-up steps by removing compounds present at high levels in extraction blanks (median samples/median blanks > 3), as well as removing compounds with relative standard deviation in the pooled samples > 30% and including compounds having a detection rate > 70% after the filtering and clean-up steps. Identification was based on in-house spectral library based on m/z, MS/MS and retention time data.

### Analysis of bile acids and PFAS

Serum samples were analyzed for bile acids (BAs) and PFAS employing modified and previously described method (Salihovic et al., 2020). Samples were randomized and 50 µL serum aliquots were extracted with 100 µL acetonitrile (Fisher Scientific, UK), with addition of 10 µL 200 µg/mL PFAS internal standards (Wellington Labortories, Canada) and 20 µL 440–670 ng/mL BA internal standards (Qmx Laboratories Ltd., UK) in methanol (Supplementary Table A1, see also BA abbreviations).

The samples were centrifuged for 3 min at 9400 × g, organic phase collected and evaporated under gentle nitrogen flow to dryness, and residue was dissolved in 300 µL 2 mM ammonium acetate (Fisher Scientific, UK) in water. After addition of 10 µL 200 µg/mL injection standard containing labelled standards (Wellington Laboratories, see Supplementary Table A1), samples were analyzed on Acquity UPLC instrument coupled to a triple quadrupole mass spectrometer (Waters Corporation, USA). A system was equipped with a perfluorinated compounds (PFC) isolator trap column installed between pump and injector, 1.7 µm 2.1 mm × 100 mm UPLC BEH C18 column and an atmospheric electrospray interface operating in negative ion mode. Mobile phases used were: 2 mM NH_4_Ac in water (A) and 9:1 methanol:2 mM NH_4_Ac in methanol (B). Following gradient program at constant 0.3 mL/min flow rate was used to separate compounds of interest: up to 1 min, 99% solvent A; 1–16 min 1% solvent A; 16–17 min 99% solvent A. Data acquisition was performed in a multiple reaction monitoring (MRM) mode. Six points external calibration curve at 0.5–160 ng/mL concentration range was used for the quantification of BAs purchased from Sigma-Aldrich, Germany; Steraloids, USA; Calbiochem, USA; and Fluka Chemie, Switzerland.

### Polar metabolites analysis

Protein precipitation was performed with a previously described procedure (Castillo et al., 2011). Aliquots of 30 µL serum were extracted with 400 µL of MeOH containing 1 µg/mL internal standards from Sigma-Aldrich, Germany (Supplementary Table A1). Samples were then vortexed and kept on ice for 30 min, prior to the centrifugation for 3 min at 9400 × g. Supernatants with a volume of 350 µL were transferred to new vials and evaporated to dryness under nitrogen flow. Two-step derivatization was performed by incubation for 60 min at 45 °C at each step, first with 25µL 20 mg/mL metoxamine (MOX) in pyridine and secondly with 25µL n-methyl-n-trimethylsilyl-trifluoroacetamide (MSTFA). A retention index (RI) standard mixture containing 10 µg/mL n-alkanes in hexane was added before the analysis on Agilent 7890B gas chromatograph coupled to 7200 Q-TOF/MS.

A programmable temperature vaporizer (PTV) injector with an initial temperature 70 °C and heated at 120 °C/min to 300 °C was used to inject 1 µL of sample at 100:1 split ratio. A Zebron ZB-SemiVolatiles column (Phenomenex, USA) with 20 m length, 0.18 mm i.d. and 0.18 µm film thickness and 1.2 mL/min helium (Linde Gas AB, Sweden) were used. Oven temperature program was as follows: after 5 min at initial 50 °C, temperature was first raised at 20 °C/min to 270 °C, and then to 300 °C at 40 °C/min with 5 min hold time of the final temperature and 2.4 ml/min helium flow to condition the column. Electron impact (EI) source with 3 min solvent delay, set to 250 °C, 70 eV electron energy and 35 µA emission was used to ionize compounds. Data was acquired by MassHunter software applying mass range of 55–650 amu, 5 spectra/s acquisition rate, 150 °C quadrupole temperature and 1.5 mL/min nitrogen collision gas flow.

The prepared six-point external calibration curve at 0.1–80 µg/mL contained standards purchased from Sigma-Aldrich (Supplementary Table A1). Extraction blanks, pooled aliquots of samples, NIST SRM 1957 certified serum and pooled in-house samples were analyzed to control quality of the performed analysis. Analysis reproducibility was within accepted analytical methods and RSD values of metabolite concentrations in pooled NOD mouse serum samples were lower than 8%. Identification was based on comparison with authentic standards (level 1 identification) or comparison with spectral libraries (NIST2017), using also information on retention index data.

### Statistical analyses

Only metabolites detected in more than 70% of samples were selected. All datasets were then preprocessed in three steps prior to the statistical analyses. At first, values below detection limits were imputed as half the minimum-detected concentrations of the corresponding compound. Secondly, all concentrations were base-2 logarithmically transformed. Finally, all log2-transformed variables were autoscaled, *i.e.*, normalized to zero mean and unit variance.

Analysis of variance (ANOVA) and Tukey’s honest significant differences (HSD) test were generated using Microsoft Excel (Microsoft Corporation, WA, USA). Boxplots were plotted in MATLAB R2018b software (Mathworks Inc., MA, USA). The R statistical programming language and corrplot R package ver.084 were used to calculate and visualize correlations between metabolites and PFAS in NOD mouse blood serum. Principal component analysis (PCA), heatmap analysis and pathway analysis were performed using Metaboanalyst ver 5.0 web-based metabolomics data analysis tool (Xia & Wishart, 2011). The Venn diagram was plotted using the jvenn plug-in (Bardou et al., 2014).

## Results

### Metabolic profiles of NOD mice

A total 251 metabolites were identified and quantified in 22 NOD mouse blood serum samples (178 lipids, 52 polar metabolites and 21 bile acids). The major lipid classes analyzed were cholesteryl esters (CEs), ceramides (Cers), lysophosphatidylcholines (LPCs), phosphatidylcholines (PCs), phosphatidylethanolamines (PEs), sphingomyelins (SMs) and triglycerides (TGs). Polar metabolites included amino acids, free fatty acids, hydroxy acids and metabolites from central carbon metabolism. One-way ANOVA and Tukey’s HSD tests for post-hoc analysis indicated dysregulation of 14 metabolites in low exposure and 120 metabolites in high exposure NOD mouse groups (Supplementary Table A2).

### PFAS exposure associated with changes in metabolome

PFAS levels in the exposed mice (Table 2) varied from low ppb to ppm levels, with low levels of PFAS detected also in the control mice.

**Table 2:** Group average PFAS concentrations with 95% CI detected in NOD mouse blood serum

Compound	Controls (n = 8) (ng/mL)	Low exposure (n = 7) (ng/mL)	High exposure (n = 7) (ng/mL)
PFHxS	2.73 ± 0.66	411.43 ± 19.90	2215.74 ± 139.38
PFOS	2.01 ± 0.48	469.76 ± 27.50	1800.36 ± 132.38
PFOA	4.33 ± 0.74	691.36 ± 25.21	4529.93 ± 297.67
PFNA	3.06 ± 0.89	1052.48 ± 69.52	11,924.59 ± 854.80
PFDA	2.44 ± 1.08	678.5 ± 43.59	2254.6 ± 174.82
PFUnDA	1.01 ± 0.31	256.1 ± 19.20	1157.63 ± 97.81

**Fig. 1:**
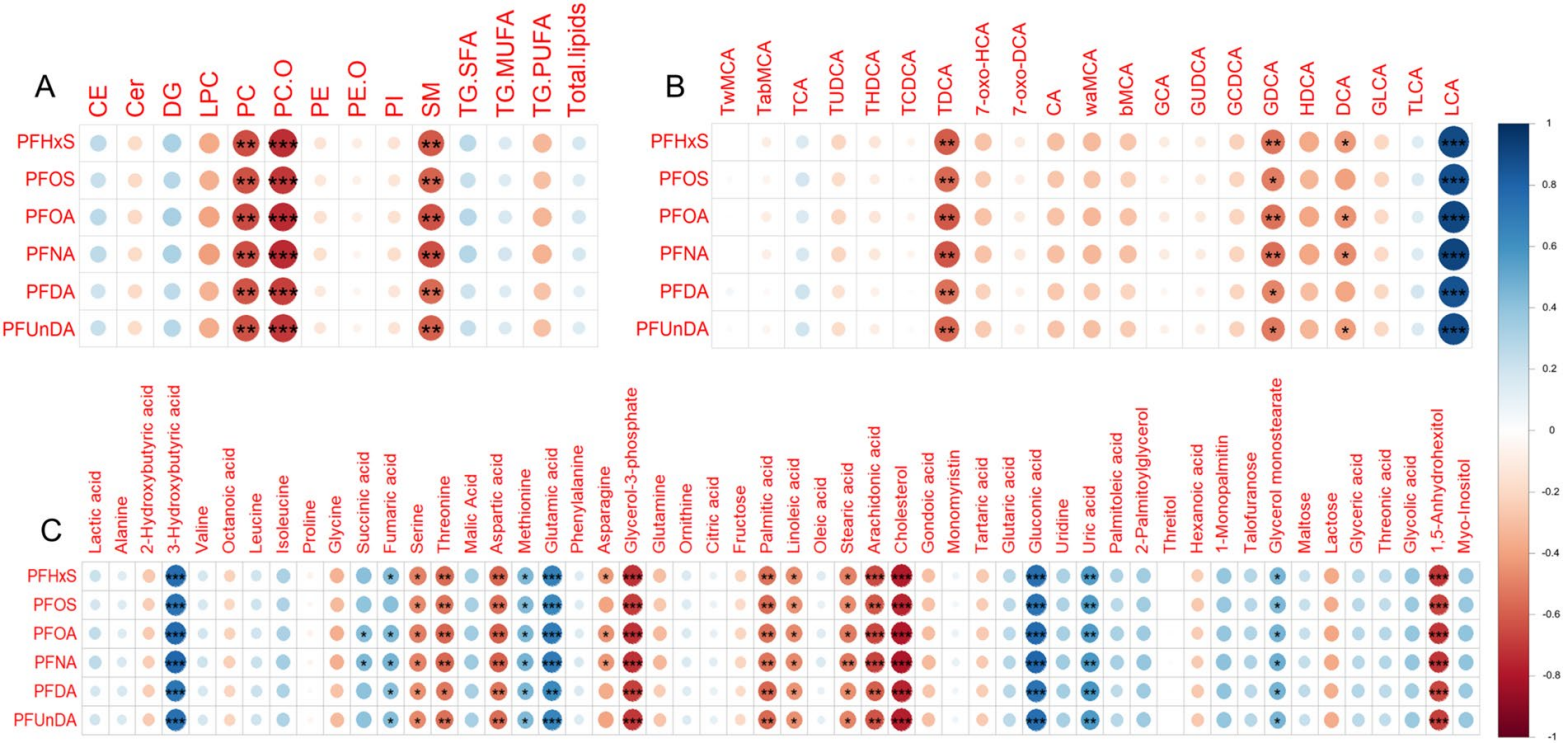
Spearman’s correlations between PFAS and lipid classes (**A**), bile acids (**B**) polar metabolites (C), including values from all 22 NOD mouse blood serum samples. Significant correlations marked with *p < 0.05, **p < 0.01 and ***p < 0.001. Positive correlation showed in blue and negative in red

ANOVA with post-hoc Tukey’s HSD tests showed that several bile acids were dysregulated in the exposed groups. Litocholic acid (LCA) was upregulated 2.1-fold (p < 0.01) in the low exposure and 5.9-fold (p < 0.001) in the high exposure versus the control group. In contrast, hyodeoxycholic acid (HDCA) was 4.3-fold downregulated in the high exposure group compared to the controls, while 11 BAs were significantly downregulated in the high versus the low exposure group (Supplementary Table A2). Spearman’s correlation analysis revealed that two bile acids, namely taurodeoxycholic acid TDCA (p < 0.01) and glycodeoxycholic acid GDCA (p < 0.05), were negatively correlated, while LCA (p < 0.001) was positively correlated with PFAS in NOD mouse blood serum samples (Fig. 1). In addition, most of BAs showed tendency of negative correlation to PFAS.

In lipids, the most prominent exposure-induced changes were observed among the LPC, PC and SM lipid classes (Fig. 1). Spearman’s correlation analysis showed that LPC 16:0e (p < 0.001), LPC 16:0p (p < 0.001), LPC 18:2 (p < 0.05), LPC 20:4 (p < 0.05), LPC 22:5 (p < 0.001) and LPC 22:6 (p < 0.05) were inversely, while LPC 20:3 (p < 0.01) was positively correlated with PFAS concentrations. Out of 67 PCs, 46 were negatively and two positively associated with PFAS in mouse serum. All except one of fourteen SMs had inverse correlation with PFAS. Additionally, four TGs correlated with PFAS exposure.

Out of 52 polar metabolites, three, 19 and 16 metabolites showed statistically-significant changes (p < 0.05) in low exposure *vs.* control, high exposure *vs.* control and high exposure *vs.* low exposure groups respectively (Supplementary Table A2).

Correlation between PFAS and polar metabolites, such as amino acids, free fatty acids, short chain fatty acids showed inverse correlation (Fig. 1) of serine (p < 0.05), threonine (p < 0.01), aspartic acid (p < 0.01), glycerol-3-phosphate (p < 0.001), palmitic acid (p < 0.01), linoleic acid (p < 0.05), stearic acid (p < 0.05), arachidonic acid (p < 0.01), cholesterol (p < 0.001) and tentatively identified 1,5-anhydrohexitol (p < 0.001). 3-hydroxybutyric acid (p < 0.001), fumaric acid (p < 0.05), methionine (p < 0.05), glutamic acid (p < 0.001) and tentatively-identified gluconic acid (p < 0.001), uric acid (p < 0.01) and glycerol monostearate (p < 0.05) were positively correlated with PFAS levels.

**Fig. 2:**
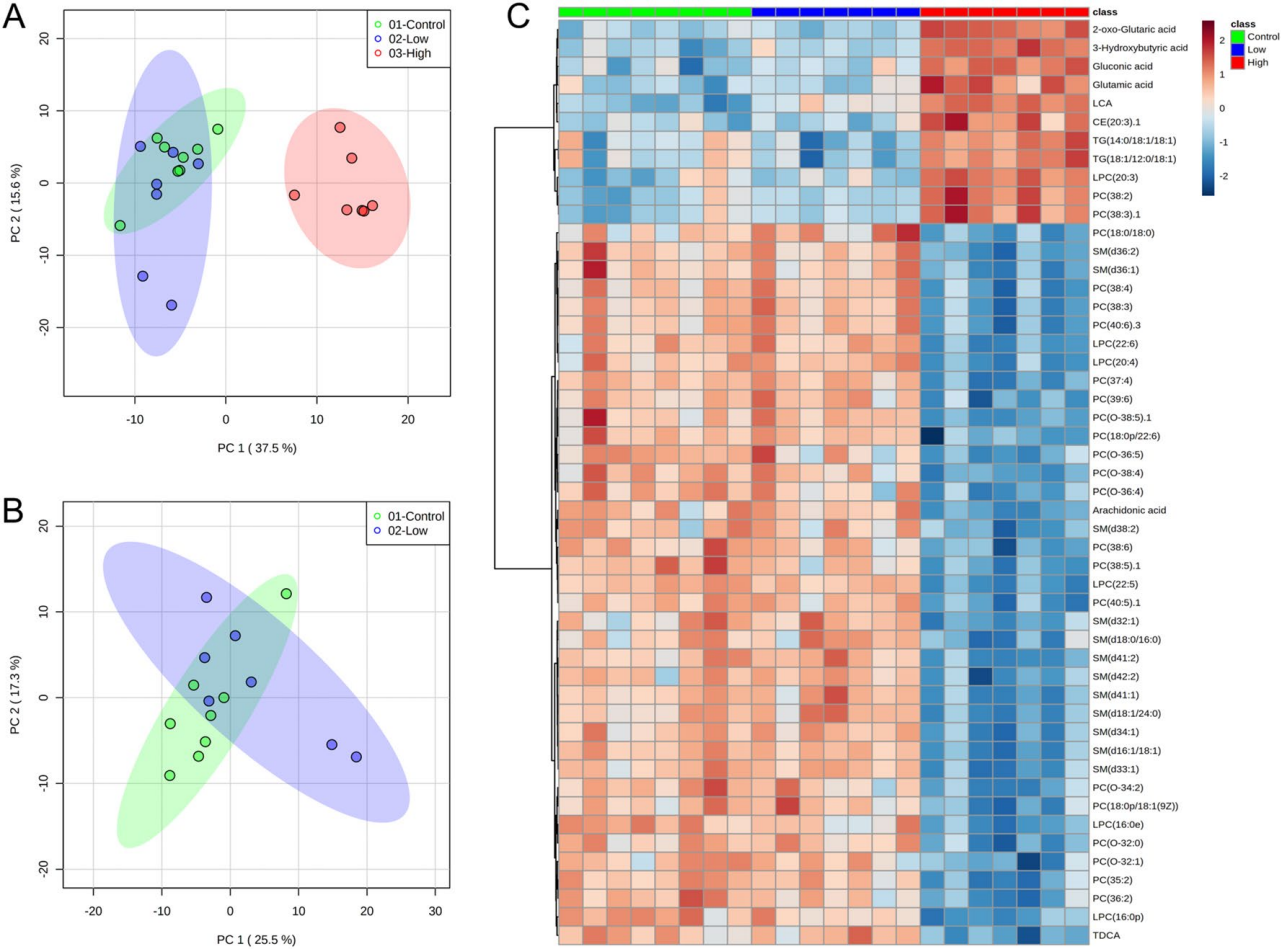
**A** and **B** PCA plots of metabolic profiles in NOD mice after exposure to a POP mixture for control (green), low exposure (blue) and high exposure (red) groups **C** Heatmap of 50 significantly-altered metabolites (ANOVA, p < 0.05) with the most contrasting patterns. Samples are sorted by the exposure group and metabolites clustered based on Ward’s clustering algorithm.

Principal component analysis (PCA) showed that the metabolic profile in the high exposure group was clearly different to that of the control and low exposure group (Fig. 2A). With the first three principal components (PC) included, the explained cumulative variance of the model was 62.1%. However, there was no clear difference between controls and low exposure group. To confirm this tendency, a PCA model only for the control and low exposure groups was created, but no distinct boundary was observed between the two groups (Fig. 2B). A heatmap of 50 metabolites with the most contrasting patterns (ANOVA, p < 0.05) illustrates these findings (Fig. 2C). A clear, exposure-dependent metabolic dysregulation was observed: polar metabolites, including glutaric, gluconic and glutamic acids, as well as the bile acid LCA were upregulated in NOD mice exposed to a higher dose of POP mixture, while many SMs, PCs and LPCs were downregulated.

To investigate metabolites that were dysregulated already in the low exposure group, only in high exposure group and in both groups, a Venn diagram (Supplementary Figure A1) was plotted using jvenn plug-in (Bardou et al., 2014). Over one hundred metabolites (138) were significantly dysregulated (Tukey’s HSD, p < 0.05) in one or two exposure groups. Eighty-seven metabolites were commonly dysregulated in high compared to low exposure group and controls (Supplementary Table A3). Five metabolites common to all three comparison groups and five other metabolites common to both exposure groups were all altered. Thus, a Venn diagram confirmed that homeostasis was maintained to a much higher degree when NOD mice were exposed to a lower dose of POP mixture.

### Interaction between BAs and other metabolites

**Fig. 3:**
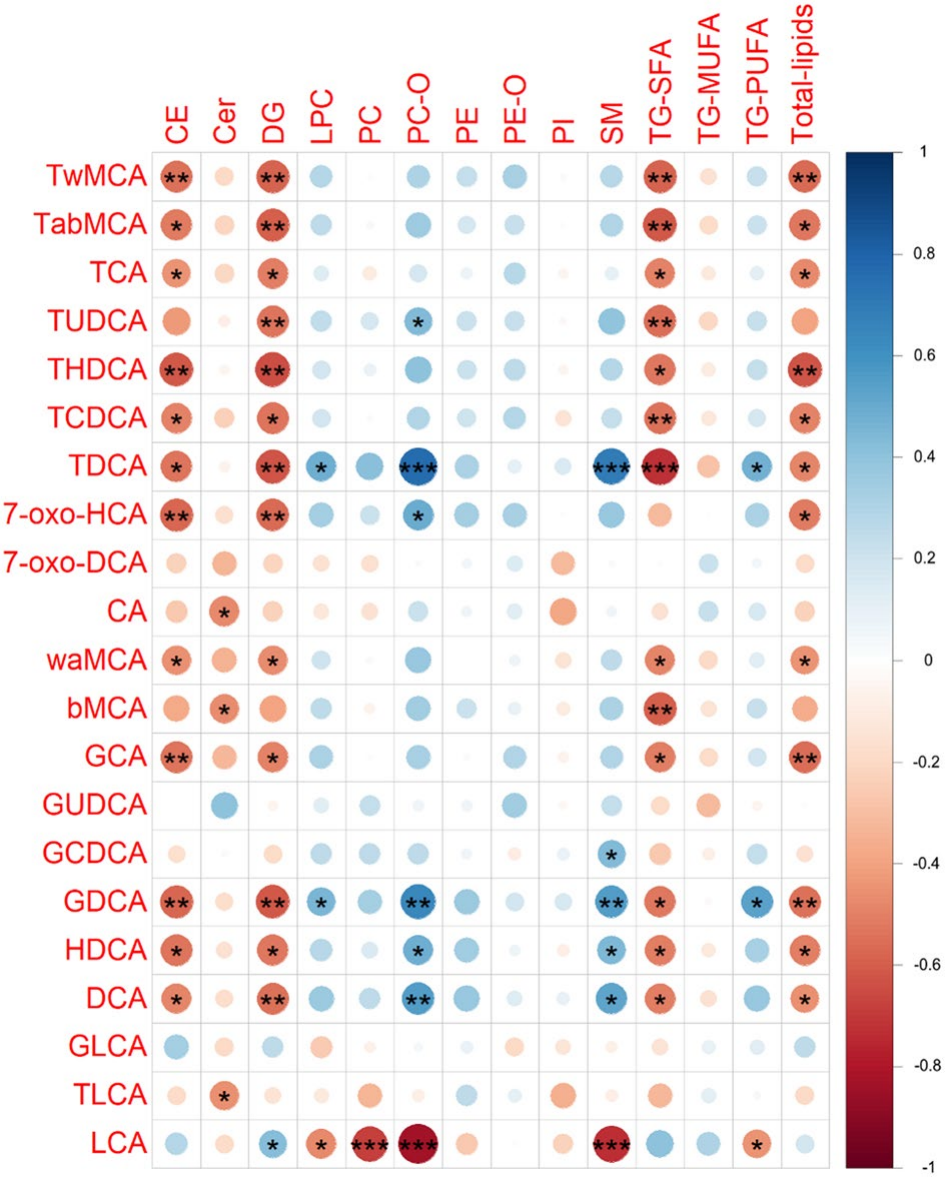
Spearman’s correlations between bile acids and lipid classes in NOD mouse blood serum samples. Significant correlations marked with *p < 0.05, **p < 0.01 and ***p < 0.001

Next, we studied the bile acid – metabolome interactions. While the majority of the BAs showed a negative correlation pattern with multiple lipid classes, the Spearman’s correlation indicated that a secondary bile acid, LCA, was positively associated with ceramides, diacylglycerol and saturated triglycerides, and negatively associated with phosphatidylcholines, lysophosphatidylcholines, sphingomyelins and polyunsaturated triglycerides (Fig. 3).

Among the polar metabolites, aspartic, stearic and arachidonic acids had the strongest positive correlation with several secondary bile acids, such as deoxycholic acid (DCA) and its conjugates. Conversely, LCA showed inverse correlation to cholesterol, amino acids and free fatty acids, including serine, threonine, and aspartic, stearic and arachidonic acids, while 3-hydroxybutyric, fumaric, gluconic and glutamic acids were positively correlated with LCA (Supplementary Figure A2).

### Pathway analysis

A pathway analysis, carried out in Metaboanalyst 5.0 using the *Mus musculus* Kyoto encyclopedia of genes and genomes (KEGG) pathway library and discrete classification, revealed 15 significantly-altered metabolic pathways (p < 0.05) (Table 3).

**Table 3:** Altered metabolic pathways in the high exposure NOD mouse group compared with controls. Only pathways with three or more matched metabolites are shown

Pathway name	Match status	p value	FDR	Database
Lipids				
Glycerophospholipid metabolism	4/36	3.51E-06	3.58E-05	KEGG
Sphingolipid metabolism	3/21	4.30E-04	1.37E-03	KEGG
Glycerolipid metabolism	3/16	7.64E-04	1.86E-03	KEGG
Bile acids				
Primary bile acid biosynthesis	7/46	7.97E-03	1.31E-02	KEGG
Polar metabolites				
Arginine biosynthesis	5/14	1.83E-06	3.11E-05	KEGG
Butanoate metabolism	3/15	9.12E-06	7.75E-05	KEGG
Aspartate Metabolism	3/34	1.06E-04	6.02E-04	SMPDB
Alanine, aspartate and glutamate metabolism	8/28	1.74E-04	7.40E-04	KEGG
Glutathione metabolism	3/28	2.77E-04	1.01E-03	KEGG
Biosynthesis of unsaturated fatty acids	5/36	3.94E-04	1.34E-03	KEGG
Glyoxylate and dicarboxylate metabolism	6/32	4.97E-04	1.49E-03	KEGG
Mitochondrial Electron Transport Chain	3/15	9.66E-04	2.39E-03	SMPDB
Arginine and proline metabolism	3/38	1.37E-03	2.91E-03	KEGG
Aminoacyl-tRNA biosynthesis	14/48	3.15E-03	6.29E-03	KEGG
Glycine, serine and threonine metabolism	4/34	4.78E-03	8.71E-03	KEGG
Valine, leucine and isoleucine biosynthesis	4/8	3.04E-02	4.37E-02	KEGG
Citrate cycle (TCA cycle)	3/20	3.08E-02	4.37E-02	KEGG

**Fig. 4:**
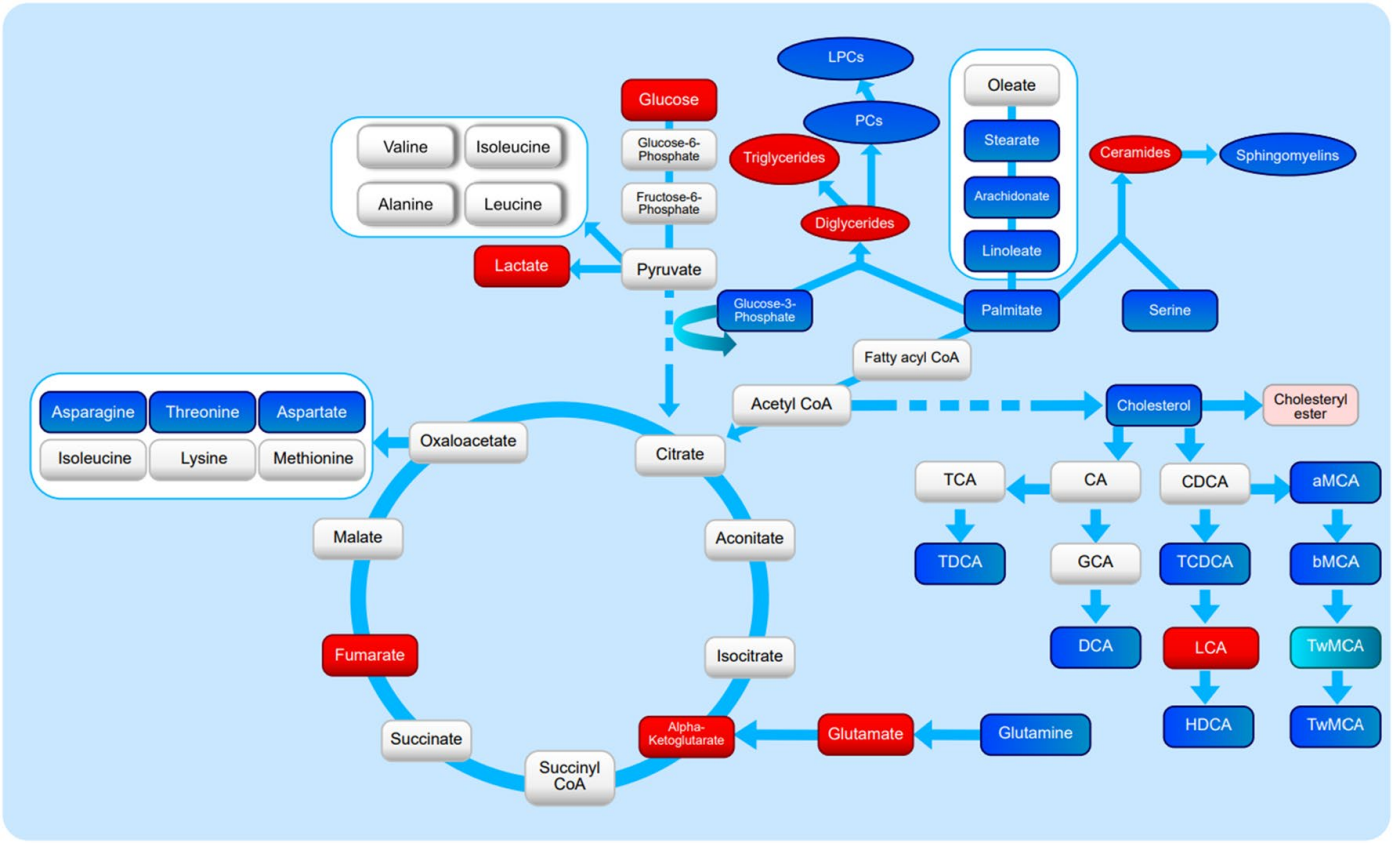
Pathway analysis diagram representing, significantly-elevated metabolites/lipid classes in red (p < 0.05) and pink (0.05 < p < 0.1), as well as downregulated metabolite groups in blue (p < 0.05) and light blue (0.05 < p < 0.1), in mice after exposure to POPs Metabolites showing no significant changes are marked in light gray

A graphic representation of altered pathways (Fig. 4) shows metabolic connections between the following downregulated classes of metabolites: (a) α-amino acids derived from oxaloacetate, (b) saturated and polyunsaturated long chain fatty acids, (c) phosphatidylcholines, (d) lysophosphatidylcholines, (e) sphingomyelins and (f) bile acids (except for increased LCA). At the same time, metabolites involved in the tricarboxylic acid cycle, glucose metabolism, and triglycerides were elevated.

## Discussion

In the present study, we investigated profiles of polar metabolites, bile acids and lipids in female NOD mice that were pre- and postnatally exposed to a POP mixture containing PCBs, OCPs, BFRs and PFAS. Exposure to the highest dose tested of these organic pollutants caused more prominent alterations in metabolic pathways associated with bile acid, glucose, TCA, fatty acid, glycerophospholipid, sphingolipid and glycerolipid metabolism compared to the lower dose administered.

This study also had some limitations. The relatively low number of mice in control, low- and high-exposure groups (8, 7 and 7 respectively) may be seen as one of the limitations of this study. However, in animal studies this number of animals is usually considered sufficient, especially due to current recommendations of minimizing the number of animals. Another study limitation is that the metabolism of mice is not fully reflecting the metabolism in humans, therefore the results of this study cannot be extrapolated directly to human metabolism even if we observed similar alterations in humans (McGlinchey et al., 2020). Another limitation is that only female NOD mice were used in the exposure study. The female NOD mice develop spontaneous disease also without any exposure and thus, comparison of diabetic versus non-diabetic animals was not feasible in this study set-up.

The POP mixture used in the present study contained a mixture of POPs at concentration ratios mimicking the exposure profiles in humans (Berntsen et al., 2017). Although the POP concentrations used in this exposure study were higher than human estimated daily intake (EDI) values, the actual circulating PFAS concentrations in the serum of the NOD mice were in the range of human exposure levels. Even in the highest exposure group, PFAS concentrations were comparable with detected levels in occupational exposure studies (Zhou et al., 2014). It should be noted that in the control group not intentionally exposed to the POPs, low levels of PFAS could be detected, possibly due to presence of PFAS in the cage- and bedding materials, dust, or water dispenser.

The terminal half-lives for PFOS and PFOA differ between days for mice (Chang et al., 2012; Lou et al., 2009) and years for humans (Bartell et al., 2010; Brede et al., 2010; Li et al., 2020b; Olsen & Zobel, 2007). Due to these large differences in metabolic processes, including liver metabolism of xenobiotics between humans and rodents (Walton et al., 2001) and the rapid xenobiotic metabolism in mice compared to humans (Martignoni et al., 2006), it was necessary to apply high initial exposure levels. Yet, daily exposure doses of individual POPs for both low and high exposure NOD mouse groups were generally lower than previously established no adverse effect levels (NOAEL) for single compounds and total concentration of organochlorines in both exposure groups was below the levels that previously was shown to induce toxicity in standard toxicity tests (Bowers et al., 2004; Darnerud, 2003; DeWitt et al., 2019).

In our study, POP exposure in NOD mice was associated with decreased levels of several phospholipids, including particularly LPCs, PCs and SMs. Similar lipid changes have been reported in children who later developed islet autoimmunity (Johnson et al., 2019) and T1D (Oresic et al., 2008). In a recent study, downregulated SMs were associated with renal impairment and all-cause mortality in T1D (Tofte et al., 2019). In animal models, elevated LPC levels have also been observed in insulin autoantibody-positive female NOD mice that did not develop diabetes compared with progressors (Sysi-Aho et al., 2011). Interestingly, LPCs have been reported to lower blood glucose levels and activate adipocyte glucose uptake in T1D and T2D mouse models (Yea et al., 2009), also enhancing insulin secretion (Soga et al., 2005).

Another prominent lipid profile change observed in this study was elevated TG levels in the exposed mouse groups, especially in TGs containing saturated or monosaturated fatty acyls. These alterations are also consistent with previous studies reporting increased plasma triglyceride levels in patients with poor glycemic control of T1D (Dullaart, 1995). Hypertriglyceridemia in T1D develops due to increased levels of circulating free fatty acids, which in turn promote a production of very low-density lipoporotein (Nikkila & Kekki, 1973). The Environmental Determinants of Diabetes in the Young (TEDDY) study also showed a decreased abundance of unsaturated triglycerides prior to appearance of autoantibodies (Li et al., 2020c). In a NOD mice study, non-treated animals which became diabetic had significantly increased levels of 3-hydroxybutyric acid, triglycerides and cholesterol compared to a group treated with immunosuppressive rapamycin, a drug that prevented the onset of T1D (Baeder et al., 1992). This is in agreement with our findings, which showed elevated concentrations of TGs and 3-hydroxybutyric acid in the exposed mice compared to a control group, indicating that exposure led to even further raised TG levels, and hypothetically could promote an earlier onset of T1D. Early signs of accelerated T1D development in these mice have also been reported, as indicated by reduced numbers of tissue-resident macrophages in pancreatic islets and a trend of reduced macrophage phagocytosis (Berntsen et al., 2018). Thus, exposure to the POP mixture reducing LPCs and SMs and increasing TG blood levels may be linked with the accelerated development of T1D in NOD mice, and the present in vivo study supports causality of previously observed association studies in humans.

We also observed changes in bile acid metabolism, with the majority of BAs being downregulated by POP exposure. The downregulation of BAs is likely to be due to the PFAS, as it has been reported both in animal models and in vivo studies as a suppressor of de novo synthesis of BAs via suppression of cholesterol 7 alpha-hydroxylase (CYP7A1), which controls the first, rate-limiting step in the formation of BAs from cholesterol through the primary pathway (Beggs et al., 2016; Behr et al., 2020; Schlezinger et al., 2020). The only upregulated BA was LCA, a secondary bile acid. Bile acids have an important role in the regulation of lipid and glucose metabolism. Given this, we investigated whether the changes in the bile acid pool due to exposure were associated with changes in lipid metabolism. Indeed, we observed that LCA had a very strong positive correlation with those lipids that are classified as lipotoxic, namely, CEs, DGs and TGs containing saturated fatty acyls, while showing strong negative association with SMs and ether phosphatidylcholines. LCA has been previously associated with hepatotoxicity and decreased LPC and SM levels in mice (Matsubara et al., 2011), and also with the alteration of gene expression involved in cholesterol and phospholipid homeostasis, leading to an inflammation and liver injury (Dionne et al., 1994). A counterintuitive dysregulation pattern where 11 BAs were dysregulated in high exposure versus low exposure groups compared to only two dysregulated bile acids in high exposure vs control group may be explained by the observation that in the low exposure group, BAs showed upregulation trend, whereas in the high exposure group they were significantly downregulated (Supplementary Figure A3), suggesting a non-monotonic response pattern. Observed alteration of bile acids in our study may explain the dysregulation of specific lipid classes that were associated with T1D development in human studies (McGlinchey et al., 2020; Oresic et al., 2008).

Changes in the overall NOD mice bile acid pool were also observed. Both conjugated secondary (GDCA, GUDCA, TDCA, TDHCA, TLCA, TUDCA) and primary (GCA, GCDCA, TabMCA, TCA, TCDCA, TwMCA) bile acids were downregulated, with the decrease of conjugated secondary BAs being more pronounced (Supplementary Figure A3). The decrease of secondary BAs produced from the primary bile acids by colonic bacteria may suggest an alteration in the gut microbiota due to the dietary POP exposure via food pellets. Indeed, earlier findings have shown that POP exposure is associated with decreased gut microbiome diversity (Iszatt et al., 2019).

We also observed elevated glucose and glycolic acid in the exposed mice, while only two tricarboxylic acid cycle metabolites were significantly increased, which indicate changes in central carbon metabolism. Glucose has previously demonstrated a regulation of the farnesoid X receptor expression in liver and caused alterations in lipid and bile acid metabolism in patients with insulin resistance or diabetes (Duran-Sandoval et al., 2004). Previous studies have also associated prenatal POP exposure with adiposity and disturbances in glucose metabolism (Thomas et al., 2007). Particularly, PFOA has been found to be associated with higher levels of glycerol and 30-min glucose levels during an oral glucose tolerance test and PFAS with altered fatty acid and lipid metabolism (Chen et al., 2020). However, it should be noted that NOD mice develop insulin resistance even in the absence of high circulating glucose concentrations (Chaparro et al., 2006).

## Conclusions

In conclusion, our mouse study showed that POP mixture exposure and, in particular measured blood PFAS levels, associated with alteration of multiple metabolic pathways. The metabolic changes observed were similar to those previously reported in human cohort studies investigating associations between exposure and biomarkers, including upregulation of glucose metabolites, triglycerides and lithocholic acid, and downregulation of long chain fatty acids, and several lipid classes including PCs, LPCs and SMs. Yet, the inconsistent findings in animal and epidemiological studies call for further investigations to better understand the associations between PFAS exposure and T1D development, including the underlying mechanisms. Combination of exposure studies in in vivo and in vitro models are needed to characterize the mechanisms, including investigation of metabolic changes not only in circulation but also in the liver and in feces. The latter is crucial as the exposure can have both direct impact of metabolism, but also an indirect impact via gut microbiota and gut microbial metabolites. In summary, our results suggest that observed metabolic alterations caused by exposure to the POP mixture can be used as potential biomarkers for T1D progression in exposure related studies.

## Supplementary Information

Below is the link to the electronic supplementary material.

Supplementary file1 (DOCX 241 kb)
