# Supplementary material for: Exposure to persistent organic pollutants alters the serum metabolome in non-obese diabetic mice
Source: Metabolomics. 2022 Nov 3;18(11):87. doi: 10.1007/s11306-022-01945-0 (PMC9633531; doi:10.1007/s11306-022-01945-0)
Supplement: Supplementary file 1 — Supplementary file1 (DOCX 241 kb) [file 11306_2022_1945_MOESM1_ESM.docx]

Supplementary tables and figures

**8.1 Tables**

**Supplementary Table A1.** Compounds used in calibration curves and internal standard mixtures.

| **Lipidomics** | **BAs and PFAS** | **Polar metabolites** |
| --- | --- | --- |
| *Calibration standards*  CE(18:0)  CE(18:2)  Cer(d18:0/18:1(9Z))  DG(18:0/18:2)  LPC(16:0)  LPC(18:0)  LPC(18:1)  LPE(18:1)  PC(16:0e/18:1(9Z))  PC(18:0p/18:1(9Z))  PC(18:0p/22:6)  PE(16:0/18:1)  PE(16:0/18:1(9Z))  TG(16:0/16:0/16:0)  TG(18:0/18:0/18:0) | *Calibration standards*  7-oxo-hyocholic acid (7-oxo-HCA)  7-oxo-deoxycholic acid (7-oxo-DCA)  alpha-Muricholic acid (αMCA)  beta-Muricholic acid (βMCA)  omega-Muricholic acid (ωMCA)  Cholic acid (CA)  Deoxycholic acid (DCA)  Glycocholic acid (GCA)  Glycochenodeoxycholic acid (GCDCA)  Glycodeoxycholic acid (GDCA)  Glycolitocholic acid (GLCA)  Glycoursodeoxycholic acid (GUDCA)  Hyodeoxycholic acid (HDCA)  Litocholic acid (LCA)  Tauro-alpha-muricholic acid (TαMCA)  Tauro-beta-muricholic acid (TβMCA)  Tauro-omega-muricholic acid (TωMCA)  Taurocholic acid (TCA)  Taurochenodeoxycholic acid (TCDCA)  Taurodeoxycjolic acid (TDCA)  Taurohyodeoxycholic acid (THDCA)  Taurolitocholic acid (TLCA)  Tauroursodeoxycholic acid (TUDCA)  Perfluorooctanoic acid (PFOA)  Perfluorononanoic acid (PFNA)  Perfluorodecanoic acid (PFDA)  Perfluoroundecanoic acid (PFUnDA)  Perfluorohexanesulfonic acid (PFHxS)  Perfluorooctanesulfonic acid (PFOS) | *Calibration standards*  Alanine  Asparagine  Aspartic acid  Glutamic acid  Glutamine  Glycine  Isoleucine  Leucine  Methionine  Ornithine  Phenylalanine  Proline  Serine  Threonine  Valine  2-hydroxybutyric acid  3-hydroxybutyric acid Arachidonic acid  Citric acid  Fumaric acid  Isocitric acid  Lactic acid  Linoleic acid  Malic acid  Octanoic acid  Oleic acid  Palmitic acid  Stearic acid  Succinic acid  Cholesterol  Fructose  Glycerol-3-phosphate |
| *Internal standards*  Cer(d18:1/17:0)  LPC(17:0)  PC(16:0/d31/18:1)  PC(17:0/17:0)  PE(17:0/17:0)  SM(d18:1/17:0)  TG(17:0/17:0/17:0) | *Internal standards*  [^13^C4]-PFPeA  [^13^C2]-PFHxA  [^13^C4]-PFHpA  [^13^C4]-PFOA  [^13^C5]-PFNA  [^13^C2]-PFDA  [^13^C2]-PFUnDA  [^13^C2]-PFDoDA  [^13^C3]-PFBS  [^18^O3]-PFHxS  [^13^C4]-PFOS  [^2^D4]-CDCA  [^2^D4]-CA  [^2^D4]-DCA  [^2^D4]-GCDCA  [^2^D4]-GCA  [D4]-GLCA  [D4]-GUDCA  [D4]-LCA  [D4]-TCA  [D4]-UDCA | *Internal standards*  Heptadecanoic acid  [^2^D5]-glutamic acid  [^2^D4]-succinic acid  [^2^D8]-valine |
|  | *Injection standards*  [^13^C5]-PFpeA  [^13^C5]-PFHxA  [^13^C8]-PFOA  [^13^C9]-PFNA  [^13^C6]-PFDA  [^13^C7]-PFUnDA  [^13^C4]-PFHxS  [^13^C8]-PFOS |  |

**Supplementary Table A2.** Level of identification based on Metabolomics Standard initiative, fold changes in median metabolite concentrations and p-values obtained after ANOVA and Tukey’s HSD post-hoc tests. Significant changes marked in green

|  | **Low/Ctrl** | | **High/Ctrl** | | **High/Low** | |
| --- | --- | --- | --- | --- | --- | --- |
| *Metabolite [level of identification]* | *Fold change* | *p-value* | *Fold change* | *p-value* | *Fold change* | *p-value* |
|  | ***Polar metabolites*** | | | | | |
| Lactic acid [1] | 0.90 | 3.49E-01 | 1.16 | 5.44E-02 | 1.29 | 2.90E-03 |
| Alanine [1] | 1.00 | 9.73E-01 | 1.07 | 9.09E-01 | 1.07 | 9.83E-01 |
| 2-Hydroxybutyric acid [1] | 1.03 | 9.17E-01 | 0.83 | 4.81E-01 | 0.80 | 7.44E-01 |
| 3-Hydroxybutyric acid [1] | 1.18 | 5.42E-01 | 3.88 | 3.20E-11 | 3.30 | 2.67E-10 |
| Valine [1] | 0.90 | 9.71E-01 | 1.11 | 4.23E-01 | 1.23 | 3.28E-01 |
| Octanoic acid [1] | 1.04 | 9.82E-01 | 0.94 | 4.73E-01 | 0.91 | 3.97E-01 |
| Leucine [1] | 1.02 | 8.59E-01 | 1.07 | 6.42E-01 | 1.05 | 9.35E-01 |
| Isoleucine [1] | 1.07 | 9.97E-01 | 1.24 | 1.58E-01 | 1.16 | 1.99E-01 |
| Proline [1] | 0.88 | 9.04E-01 | 0.89 | 9.81E-01 | 1.01 | 8.16E-01 |
| Glycine [1] | 0.99 | 9.73E-01 | 0.80 | 7.13E-02 | 0.81 | 5.34E-02 |
| Succinic acid [1] | 0.90 | 8.65E-01 | 1.96 | 1.50E-01 | 2.17 | 3.75E-01 |
| Fumaric acid [1] | 0.98 | 9.57E-01 | 1.56 | 2.68E-02 | 1.59 | 5.87E-02 |
| Serine [1] | 0.93 | 4.94E-01 | 0.75 | 2.27E-02 | 0.81 | 2.46E-01 |
| Threonine [1] | 0.97 | 6.61E-01 | 0.61 | 3.48E-03 | 0.63 | 3.22E-02 |
| Malic Acid [1] | 1.04 | 9.88E-01 | 1.42 | 2.27E-01 | 1.37 | 3.12E-01 |
| Aspartic acid [1] | 0.94 | 8.87E-01 | 0.75 | 3.76E-04 | 0.80 | 1.60E-03 |
| Methionine [1] | 1.06 | 8.15E-01 | 1.24 | 9.32E-02 | 1.17 | 3.00E-01 |
| Glutamic acid [1] | 1.07 | 8.61E-01 | 1.73 | 7.31E-07 | 1.61 | 3.57E-06 |
| Phenylalanine [1] | 0.91 | 8.55E-01 | 1.04 | 8.02E-01 | 1.14 | 9.97E-01 |
| Asparagine [1] | 1.09 | 9.79E-01 | 0.74 | 2.73E-02 | 0.68 | 4.98E-02 |
| Glycerol-3-phosphate [1] | 0.75 | 7.57E-02 | 0.57 | 1.39E-04 | 0.76 | 3.88E-02 |
| Glutamine [1] | 1.13 | 6.49E-01 | 0.52 | 8.64E-02 | 0.46 | 1.59E-02 |
| Ornithine [1] | 0.85 | 8.20E-01 | 1.09 | 5.14E-01 | 1.28 | 2.36E-01 |
| Citric and isocitric acids [1] | 1.03 | 7.61E-01 | 1.10 | 7.18E-01 | 1.07 | 3.29E-01 |
| Fructose [1] | 1.20 | 9.79E-01 | 0.70 | 3.42E-01 | 0.59 | 2.76E-01 |
| Palmitic acid [1] | 0.75 | 6.38E-03 | 0.81 | 3.84E-02 | 1.08 | 6.39E-01 |
| Linoleic acid [1] | 0.85 | 5.13E-01 | 0.62 | 4.44E-02 | 0.73 | 3.70E-01 |
| Oleic acid [1] | 0.82 | 3.58E-01 | 1.10 | 4.16E-01 | 1.34 | 3.91E-02 |
| Stearic acid [1] | 1.12 | 7.19E-01 | 0.76 | 7.90E-04 | 0.68 | 1.85E-04 |
| Arachidonic acid [1] | 0.93 | 9.76E-01 | 0.54 | 4.38E-07 | 0.58 | 1.12E-06 |
| Cholesterol [1] | 0.76 | 1.07E-02 | 0.49 | 7.84E-06 | 0.64 | 1.92E-02 |
| Gondoic acid [2] | 1.12 | 9.28E-01 | 0.84 | 1.47E-01 | 0.75 | 8.39E-02 |
| Monomyristin [2] | 0.94 | 6.56E-01 | 1.02 | 7.01E-01 | 1.09 | 2.42E-01 |
| Tartaric acid [2] | 1.30 | 9.99E-01 | 0.79 | 3.42E-01 | 0.60 | 3.50E-01 |
| Glutaric acid [2] | 1.06 | 9.45E-01 | 1.26 | 4.57E-01 | 1.19 | 3.14E-01 |
| Gluconic acid [2] | 1.27 | 3.45E-01 | 2.99 | 9.99E-10 | 2.36 | 1.92E-08 |
| Uridine [2] | 1.06 | 7.88E-01 | 1.21 | 3.70E-01 | 1.13 | 7.83E-01 |
| Uric acid [2] | 1.45 | 1.25E-01 | 1.74 | 2.00E-02 | 1.20 | 7.03E-01 |
| Palmitoleic acid [2] | 0.80 | 3.26E-01 | 1.46 | 1.48E-03 | 1.83 | 7.06E-05 |
| 2-Palmitoylglycerol [2] | 1.01 | 8.98E-01 | 1.11 | 1.82E-01 | 1.11 | 3.93E-01 |
| Threitol [2] | 0.99 | 9.96E-01 | 0.95 | 1.00E+00 | 0.96 | 9.97E-01 |
| Hexanoic acid [2] | 0.87 | 5.69E-01 | 0.89 | 6.35E-01 | 1.03 | 9.89E-01 |
| 1-Monopalmitin [2] | 1.13 | 6.47E-01 | 1.11 | 1.53E-01 | 0.98 | 6.00E-01 |
| Talofuranose [2] | 0.94 | 9.81E-01 | 1.49 | 4.28E-01 | 1.59 | 5.61E-01 |
| Glycerol monostearate [2] | 1.05 | 6.41E-01 | 1.19 | 4.13E-02 | 1.13 | 2.58E-01 |
| Maltose [2] | 0.94 | 8.20E-01 | 1.01 | 8.37E-01 | 1.07 | 9.99E-01 |
| Lactose [2] | 0.77 | 9.27E-01 | 0.54 | 1.39E-01 | 0.70 | 2.87E-01 |
| Glyceric acid [2] | 1.17 | 3.46E-01 | 0.96 | 6.70E-01 | 0.82 | 8.23E-01 |
| Threonic acid [2] | 0.94 | 8.32E-01 | 1.07 | 5.30E-01 | 1.14 | 8.84E-01 |
| Glycolic acid [2] | 0.94 | 9.85E-01 | 1.26 | 2.72E-02 | 1.34 | 2.36E-02 |
| 1,5-Anhydrohexitol [2] | 0.85 | 3.78E-02 | 0.59 | 1.63E-05 | 0.69 | 1.10E-02 |
| Myo-Inositol [2] | 1.20 | 7.28E-01 | 1.24 | 1.61E-01 | 1.03 | 5.35E-01 |
| Glucose [2] | 1.02 | 1.99E-01 | 1.27 | 2.55E-03 | 1.24 | 1.26E-01 |
|  | ***Bile acids*** | | | | | |
| TwMCA [1] | 6.79 | 1.49E-01 | 1.01 | 5.54E-01 | 0.15 | 1.96E-02 |
| TabMCA [1] | 3.57 | 4.37E-01 | 0.79 | 4.37E-01 | 0.22 | 5.73E-02 |
| TCA [1] | 6.28 | 2.26E-01 | 2.23 | 9.13E-01 | 0.36 | 1.14E-01 |
| TUDCA [1] | 3.35 | 7.41E-02 | 0.58 | 3.71E-01 | 0.17 | 4.20E-03 |
| THDCA [1] | 3.32 | 1.41E-01 | 0.27 | 6.05E-01 | 0.08 | 2.20E-02 |
| TCDCA [1] | 3.17 | 1.19E-01 | 0.70 | 4.92E-01 | 0.22 | 1.19E-02 |
| TDCA [1] | 1.55 | 8.85E-01 | 0.05 | 6.01E-02 | 0.03 | 2.69E-02 |
| 7-oxo-HCA [1] | 4.02 | 2.75E-01 | 0.20 | 3.55E-01 | 0.05 | 2.08E-02 |
| 7-oxo-DCA [1] | 2.28 | 6.60E-01 | 0.48 | 7.53E-01 | 0.21 | 2.79E-01 |
| CA [1] | 1.05 | 9.69E-01 | 0.18 | 8.32E-01 | 0.17 | 7.06E-01 |
| waMCA [1] | 3.40 | 2.22E-01 | 0.43 | 2.85E-01 | 0.13 | 1.12E-02 |
| bMCA [1] | 2.05 | 1.34E-01 | 0.08 | 1.10E-01 | 0.04 | 1.66E-03 |
| GCA [1] | 2.77 | 1.94E-01 | 0.74 | 8.17E-01 | 0.27 | 6.59E-02 |
| GUDCA [1] | 0.64 | 9.83E-01 | 0.59 | 9.69E-01 | 0.93 | 9.14E-01 |
| GCDCA [1] | 0.99 | 9.94E-01 | 0.91 | 4.91E-01 | 0.93 | 5.76E-01 |
| GDCA [1] | 1.16 | 9.81E-01 | 0.09 | 7.23E-02 | 0.08 | 1.20E-01 |
| HDCA [1] | 1.38 | 1.88E-01 | 0.23 | 2.51E-02 | 0.17 | 5.05E-04 |
| DCA [1] | 1.97 | 8.52E-01 | 0.28 | 1.18E-01 | 0.14 | 4.72E-02 |
| GLCA [1] | 0.91 | 8.02E-01 | 0.90 | 5.71E-01 | 0.99 | 9.34E-01 |
| TLCA [1] | 1.41 | 3.32E-01 | 0.66 | 7.86E-01 | 0.47 | 1.15E-01 |
| LCA [1] | 2.14 | 2.33E-03 | 5.92 | 1.44E-12 | 2.77 | 4.84E-10 |
|  | ***Lipids*** | | | | | |
| CE(16:0) [2] | 0.84 | 9.39E-02 | 0.88 | 1.08E-01 | 1.04 | 9.97E-01 |
| CE(18:1) [2] | 0.90 | 2.04E-01 | 1.02 | 9.82E-01 | 1.13 | 1.67E-01 |
| CE(18:1) [2] | 0.90 | 6.60E-02 | 0.93 | 1.58E-01 | 1.03 | 8.92E-01 |
| CE(16:0) [2] | 0.66 | 2.81E-01 | 0.95 | 5.48E-01 | 1.45 | 4.87E-02 |
| CE(18:2) [1] | 0.98 | 3.81E-01 | 1.02 | 7.84E-01 | 1.04 | 7.84E-01 |
| CE(20:3) [2] | 0.90 | 6.33E-01 | 0.74 | 3.11E-02 | 0.82 | 1.99E-01 |
| CE(20:3) [2] | 1.04 | 9.93E-01 | 2.59 | 6.88E-06 | 2.49 | 1.32E-05 |
| CE(20:3) [2] | 1.25 | 6.54E-01 | 1.76 | 8.61E-01 | 1.41 | 3.77E-01 |
| CE(18:2) [2] | 1.09 | 6.54E-01 | 2.26 | 4.51E-01 | 2.07 | 1.27E-01 |
| CE(20:4) [2] | 0.94 | 6.83E-01 | 0.74 | 4.83E-02 | 0.78 | 2.45E-01 |
| Cer(d18:1/22:0) [2] | 0.86 | 5.80E-01 | 0.89 | 3.84E-01 | 1.03 | 9.40E-01 |
| Cer(d18:1/23:0) [2] | 0.80 | 3.29E-01 | 0.90 | 9.49E-01 | 1.12 | 5.14E-01 |
| Cer(d18:1/24:0) [2] | 0.84 | 2.16E-01 | 0.72 | 2.03E-01 | 0.85 | 9.99E-01 |
| Cer(d18:1/24:1) [2] | 0.88 | 7.95E-01 | 1.20 | 2.82E-01 | 1.37 | 1.06E-01 |
| DG(18:1/18:1) [1] | 0.80 | 6.16E-01 | 2.71 | 4.11E-01 | 3.41 | 1.00E-01 |
| LPC(16:0e) [2] | 0.92 | 1.18E-01 | 0.58 | 3.15E-07 | 0.62 | 2.88E-05 |
| LPC(16:0p) [2] | 0.77 | 1.06E-01 | 0.37 | 8.79E-07 | 0.49 | 1.02E-04 |
| LPC(16:1) [2] | 0.83 | 2.09E-01 | 1.31 | 1.21E-02 | 1.58 | 3.36E-04 |
| LPC(18:0) [1] | 1.18 | 1.82E-01 | 0.88 | 8.08E-02 | 0.75 | 2.06E-03 |
| LPC(18:1) [1] | 0.89 | 4.52E-01 | 1.26 | 9.50E-03 | 1.41 | 8.43E-04 |
| LPC(18:2) [2] | 0.93 | 8.64E-01 | 0.86 | 5.16E-02 | 0.92 | 1.54E-01 |
| LPC(20:3) [2] | 1.07 | 9.71E-01 | 3.14 | 2.34E-07 | 2.94 | 2.54E-07 |
| LPC(20:4) [2] | 1.06 | 6.31E-01 | 0.53 | 1.02E-05 | 0.50 | 2.53E-06 |
| LPC(22:5) [2] | 0.98 | 3.81E-01 | 0.29 | 9.74E-10 | 0.29 | 1.25E-08 |
| LPC(22:6) [2] | 1.05 | 7.44E-01 | 0.54 | 6.02E-06 | 0.51 | 2.21E-06 |
| LPE(18:1) [1] | 0.87 | 5.44E-01 | 1.15 | 2.29E-02 | 1.32 | 2.88E-03 |
| PC(16:0/16:0) [1] | 0.89 | 1.79E-01 | 0.70 | 2.60E-04 | 0.79 | 2.01E-02 |
| PC(16:0e/18:1(9Z)) [1] | 0.99 | 7.87E-01 | 0.86 | 4.44E-02 | 0.87 | 1.73E-01 |
| PC(18:0/18:0) [1] | 1.28 | 1.17E-01 | 0.59 | 4.63E-03 | 0.46 | 6.51E-05 |
| PC(18:0p/18:1(9Z)) [1] | 1.00 | 9.79E-01 | 0.44 | 5.25E-04 | 0.44 | 4.75E-04 |
| PC(18:0p/22:6) [1] | 1.15 | 9.92E-01 | 0.31 | 2.61E-04 | 0.27 | 4.77E-04 |
| PC(30:0) [2] | 0.85 | 2.35E-01 | 0.69 | 4.23E-03 | 0.81 | 1.56E-01 |
| PC(31:0) [2] | 0.97 | 9.98E-01 | 0.47 | 6.57E-04 | 0.48 | 7.91E-04 |
| PC(32:1) [2] | 0.77 | 2.48E-02 | 1.13 | 9.27E-01 | 1.46 | 1.40E-02 |
| PC(32:2) [2] | 0.58 | 1.52E-02 | 0.59 | 6.71E-03 | 1.01 | 9.31E-01 |
| PC(33:1) [2] | 0.70 | 1.55E-02 | 0.67 | 1.76E-02 | 0.96 | 9.98E-01 |
| PC(34:1) [2] | 0.70 | 9.54E-03 | 1.14 | 1.03E-01 | 1.62 | 1.13E-04 |
| PC(34:2) [2] | 0.87 | 1.06E-02 | 0.82 | 1.23E-02 | 0.95 | 9.98E-01 |
| PC(34:3) [2] | 0.74 | 1.25E-02 | 1.10 | 9.81E-01 | 1.48 | 1.05E-02 |
| PC(35:1) [2] | 0.82 | 8.31E-02 | 0.67 | 1.83E-04 | 0.82 | 3.46E-02 |
| PC(35:2) [2] | 0.97 | 5.10E-01 | 0.50 | 3.43E-07 | 0.52 | 4.57E-06 |
| PC(35:2) [2] | 1.04 | 9.00E-01 | 0.50 | 8.86E-02 | 0.48 | 4.43E-02 |
| PC(35:3) [2] | 0.74 | 6.54E-04 | 0.84 | 1.90E-02 | 1.13 | 3.29E-01 |
| PC(35:3) [2] | 0.82 | 4.81E-01 | 0.34 | 1.66E-03 | 0.42 | 2.72E-02 |
| PC(36:1) [2] | 1.00 | 9.09E-01 | 0.74 | 2.39E-04 | 0.74 | 8.40E-04 |
| PC(36:2) [2] | 0.80 | 8.37E-02 | 0.44 | 4.11E-06 | 0.54 | 7.37E-04 |
| PC(36:2) [2] | 0.99 | 7.73E-01 | 0.75 | 1.46E-04 | 0.75 | 9.31E-04 |
| PC(36:3) [2] | 0.91 | 1.82E-01 | 1.17 | 3.52E-02 | 1.28 | 8.33E-04 |
| PC(36:4) [2] | 0.86 | 5.33E-01 | 0.79 | 1.60E-01 | 0.92 | 7.04E-01 |
| PC(36:4) [2] | 0.92 | 6.94E-02 | 0.61 | 6.55E-05 | 0.66 | 1.60E-02 |
| PC(36:4) [2] | 0.74 | 7.15E-03 | 1.19 | 3.26E-02 | 1.61 | 2.54E-05 |
| PC(36:5) [2] | 0.93 | 7.36E-01 | 0.57 | 8.82E-02 | 0.61 | 3.36E-01 |
| PC(37:1) [2] | 0.98 | 9.96E-01 | 0.56 | 7.20E-04 | 0.58 | 8.30E-04 |
| PC(37:2) [2] | 1.01 | 9.11E-01 | 0.67 | 4.08E-05 | 0.66 | 1.44E-04 |
| PC(37:3) [2] | 0.64 | 9.03E-01 | 1.06 | 7.12E-01 | 1.65 | 4.76E-01 |
| PC(37:3) [2] | 1.03 | 9.25E-01 | 0.81 | 1.61E-02 | 0.79 | 8.97E-03 |
| PC(37:4) [2] | 1.05 | 9.52E-01 | 0.29 | 1.04E-07 | 0.27 | 1.02E-07 |
| PC(38:2) [2] | 1.11 | 9.98E-01 | 2.53 | 1.07E-06 | 2.29 | 1.51E-06 |
| PC(38:3) [2] | 1.03 | 5.77E-01 | 0.51 | 1.89E-05 | 0.49 | 3.78E-06 |
| PC(38:3) [2] | 1.12 | 9.97E-01 | 2.55 | 3.06E-06 | 2.27 | 5.46E-06 |
| PC(38:4) [2] | 0.95 | 7.24E-01 | 0.44 | 7.10E-05 | 0.46 | 2.09E-05 |
| PC(38:5) [2] | 0.98 | 6.47E-01 | 0.58 | 7.26E-03 | 0.59 | 5.79E-02 |
| PC(38:5) [2] | 0.94 | 8.86E-02 | 0.37 | 8.16E-06 | 0.40 | 1.44E-03 |
| PC(38:6) [2] | 0.98 | 4.60E-01 | 0.50 | 5.62E-06 | 0.51 | 1.04E-04 |
| PC(38:6) [2] | 0.97 | 8.76E-01 | 0.68 | 4.75E-02 | 0.70 | 1.38E-01 |
| PC(39:6) [2] | 1.10 | 5.31E-01 | 0.31 | 2.00E-04 | 0.28 | 2.86E-05 |
| PC(40:4) [2] | 1.10 | 9.00E-01 | 0.77 | 9.93E-02 | 0.71 | 4.97E-02 |
| PC(40:5) [2] | 1.11 | 9.77E-01 | 0.70 | 4.61E-03 | 0.63 | 3.84E-03 |
| PC(40:5) [2] | 0.97 | 1.00E+00 | 0.32 | 4.02E-06 | 0.33 | 6.13E-06 |
| PC(40:6) [2] | 0.97 | 6.60E-01 | 0.32 | 2.73E-02 | 0.33 | 1.68E-01 |
| PC(40:6) [2] | 0.91 | 9.57E-01 | 0.45 | 9.84E-01 | 0.49 | 9.00E-01 |
| PC(40:6) [2] | 1.08 | 6.94E-01 | 0.75 | 3.92E-01 | 0.70 | 1.18E-01 |
| PC(40:6) [2] | 1.03 | 6.87E-01 | 0.58 | 1.00E-05 | 0.57 | 2.97E-06 |
| PC(40:7) [2] | 1.03 | 1.00E+00 | 0.71 | 2.33E-01 | 0.69 | 2.55E-01 |
| PC(40:7) [2] | 0.96 | 9.87E-01 | 0.49 | 9.41E-03 | 0.51 | 1.65E-02 |
| PC(40:8) [2] | 1.01 | 8.50E-01 | 0.45 | 2.88E-04 | 0.45 | 1.33E-03 |
| PC(O-32:0) [2] | 0.98 | 9.97E-01 | 0.68 | 3.61E-06 | 0.69 | 6.54E-06 |
| PC(O-32:1) [2] | 0.86 | 6.13E-01 | 0.50 | 3.17E-05 | 0.59 | 3.58E-04 |
| PC(O-34:2) [2] | 0.92 | 3.20E-01 | 0.54 | 1.81E-05 | 0.58 | 6.36E-04 |
| PC(O-34:3) [2] | 0.64 | 1.50E-01 | 0.44 | 1.15E-03 | 0.69 | 9.01E-02 |
| PC(O-36:3) [2] | 0.76 | 4.18E-02 | 0.53 | 4.11E-03 | 0.70 | 5.62E-01 |
| PC(O-36:4) [2] | 0.86 | 3.38E-01 | 0.43 | 4.58E-05 | 0.50 | 1.52E-03 |
| PC(O-36:4) [2] | 0.82 | 5.91E-01 | 0.84 | 5.15E-01 | 1.03 | 9.92E-01 |
| PC(O-36:4) [2] | 0.79 | 7.18E-01 | 0.45 | 2.28E-01 | 0.57 | 6.49E-01 |
| PC(O-36:5) [2] | 0.74 | 4.40E-01 | 0.34 | 1.80E-04 | 0.46 | 3.88E-03 |
| PC(O-38:4) [2] | 0.89 | 6.28E-01 | 0.35 | 4.39E-05 | 0.39 | 4.74E-04 |
| PC(O-38:4) or PC(P-38:3) [3] | 0.84 | 5.60E-01 | 0.43 | 4.49E-02 | 0.51 | 3.12E-01 |
| PC(O-38:5) [2] | 0.92 | 2.51E-01 | 0.60 | 3.85E-04 | 0.66 | 1.88E-02 |
| PC(O-38:5) [2] | 1.11 | 9.36E-01 | 0.49 | 4.78E-04 | 0.44 | 3.14E-04 |
| PC(O-38:6) [2] | 1.01 | 9.46E-01 | 0.44 | 1.49E-02 | 0.44 | 3.53E-02 |
| PC(O-38:6) [2] | 1.12 | 9.51E-01 | 0.48 | 1.18E-01 | 0.43 | 7.73E-02 |
| PC(O-40:6) [2] | 0.93 | 9.64E-01 | 0.17 | 8.09E-04 | 0.19 | 1.94E-03 |
| PC(P-18:0/22:6) [2] | 0.92 | 8.47E-01 | 0.52 | 1.77E-02 | 0.56 | 6.80E-03 |
| PE(16:0/18:1) [1] | 1.21 | 7.26E-01 | 0.76 | 8.49E-01 | 0.63 | 4.26E-01 |
| PE(O-16:0/22:6) or  PE(P-18:0/20:5) [3] | 0.96 | 4.22E-01 | 0.98 | 2.60E-01 | 1.03 | 9.41E-01 |
| PE(O-16:0/22:6) or  PE(P-18:0/20:5) [3] | 1.37 | 8.37E-01 | 1.82 | 5.75E-01 | 1.32 | 2.96E-01 |
| PE(O-38:5) or  PE(P-38:4) [3] | 1.12 | 9.73E-01 | 0.50 | 3.47E-02 | 0.45 | 2.66E-02 |
| PE(P-18:0/22:6) [2] | 1.09 | 8.82E-01 | 0.55 | 4.64E-03 | 0.50 | 2.13E-03 |
| PI(18:0/20:4) [1] | 1.15 | 8.40E-01 | 0.90 | 6.10E-01 | 0.78 | 3.23E-01 |
| SM(d16:1/18:1) or SM(d18:2/16:0) [3] | 1.00 | 7.86E-01 | 0.46 | 7.89E-09 | 0.46 | 3.82E-08 |
| SM(d18:0/16:0) [1] | 1.13 | 6.81E-01 | 0.61 | 4.02E-04 | 0.54 | 9.21E-05 |
| SM(d18:1/24:0) [2] | 1.10 | 2.73E-01 | 0.46 | 2.34E-05 | 0.42 | 1.53E-06 |
| SM(d32:1) [2] | 1.00 | 8.36E-01 | 0.61 | 1.14E-04 | 0.61 | 4.95E-05 |
| SM(d33:1) [2] | 1.00 | 9.96E-01 | 0.48 | 5.58E-07 | 0.48 | 7.59E-07 |
| SM(d34:1) [2] | 1.08 | 8.63E-01 | 0.61 | 2.63E-06 | 0.56 | 1.53E-06 |
| SM(d36:1) [2] | 1.12 | 9.96E-01 | 0.48 | 1.23E-03 | 0.43 | 2.02E-03 |
| SM(d36:2) [2] | 1.06 | 9.99E-01 | 0.45 | 1.66E-04 | 0.43 | 2.22E-04 |
| SM(d38:2) [2] | 0.94 | 9.18E-01 | 0.51 | 8.06E-05 | 0.54 | 2.74E-04 |
| SM(d40:1) [2] | 0.98 | 8.51E-01 | 0.74 | 1.92E-04 | 0.75 | 8.90E-04 |
| SM(d40:1) [2] | 6.03 | 2.34E-01 | 0.67 | 5.99E-01 | 0.11 | 4.58E-02 |
| SM(d41:1) [2] | 1.12 | 4.71E-01 | 0.45 | 1.29E-05 | 0.40 | 1.89E-06 |
| SM(d41:2) [2] | 1.05 | 7.92E-01 | 0.50 | 3.01E-06 | 0.48 | 1.35E-06 |
| SM(d42:2) [2] | 1.03 | 8.88E-01 | 0.40 | 6.60E-06 | 0.39 | 4.07E-06 |
| TG(14:0/16:0/18:1) [2] | 0.73 | 1.69E-01 | 2.47 | 9.55E-04 | 3.41 | 2.35E-05 |
| TG(14:0/18:1/18:1) [2] | 0.77 | 8.05E-02 | 1.91 | 6.80E-05 | 2.48 | 1.04E-06 |
| TG(14:0/18:2/18:2) [2] | 1.14 | 8.97E-01 | 2.09 | 8.04E-05 | 1.83 | 4.61E-05 |
| TG(16:0/16:0/16:0) [1] | 0.71 | 1.85E-01 | 1.28 | 5.62E-02 | 1.80 | 1.40E-03 |
| TG(16:0/18:0/18:1) [2] | 0.85 | 4.10E-01 | 1.17 | 9.62E-02 | 1.37 | 8.11E-03 |
| TG(16:0/18:2/18:2) [2] | 0.97 | 8.46E-01 | 1.04 | 5.32E-01 | 1.07 | 2.74E-01 |
| TG(16:0/18:2/18:3) [2] | 1.11 | 9.26E-01 | 1.23 | 1.28E-01 | 1.12 | 2.62E-01 |
| TG(16:0/22:5/18:1) or TG(20:4/18:1/18:1) [3] | 1.06 | 8.92E-01 | 0.67 | 1.36E-01 | 0.64 | 6.71E-02 |
| TG(18:1/12:0/18:1) or TG(18:2/16:0/14:0) [3] | 0.69 | 1.58E-01 | 2.38 | 3.95E-05 | 3.43 | 1.30E-06 |
| TG(18:1/18:1/16:0) [2] | 0.80 | 1.86E-01 | 1.66 | 2.97E-03 | 2.09 | 7.53E-05 |
| TG(18:1/18:1/18:1) [2] | 1.04 | 5.22E-01 | 1.53 | 1.12E-02 | 1.47 | 1.29E-03 |
| TG(18:1/18:1/22:6) [2] | 1.23 | 6.47E-01 | 0.43 | 2.70E-02 | 0.35 | 4.93E-03 |
| TG(18:1/18:2/18:2) [2] | 0.68 | 5.61E-01 | 1.38 | 3.78E-01 | 2.02 | 7.55E-02 |
| TG(18:2/18:1/16:0) [2] | 0.97 | 3.35E-01 | 1.14 | 1.37E-01 | 1.17 | 8.92E-03 |
| TG(18:2/18:1/18:1) [2] | 0.89 | 6.20E-01 | 1.33 | 2.95E-01 | 1.49 | 6.53E-02 |
| TG(18:2/18:2/18:2) or TG(18:3/18:2/18:1) [3] | 0.86 | 9.42E-01 | 1.89 | 1.83E-01 | 2.19 | 1.16E-01 |
| TG(18:2/18:2/18:2) or TG(18:3/18:2/18:1) [3] | 1.11 | 9.89E-01 | 4.78 | 4.48E-02 | 4.31 | 4.01E-02 |
| TG(18:2/22:5/16:0) [2] | 1.34 | 5.97E-01 | 0.57 | 8.23E-02 | 0.43 | 1.35E-02 |
| TG(47:0) [2] | 0.96 | 8.60E-01 | 0.97 | 8.55E-01 | 1.02 | 1.00E+00 |
| TG(48:0) [2] | 0.81 | 3.13E-01 | 1.35 | 6.56E-02 | 1.67 | 3.46E-03 |
| TG(48:1) [2] | 0.94 | 2.97E-01 | 2.62 | 3.87E-04 | 2.79 | 2.12E-05 |
| TG(48:3) [2] | 0.84 | 6.35E-01 | 2.19 | 2.16E-04 | 2.61 | 4.39E-05 |
| TG(49:0) [2] | 0.83 | 4.94E-02 | 0.87 | 9.95E-02 | 1.04 | 9.35E-01 |
| TG(49:1) [2] | 0.80 | 2.81E-01 | 0.91 | 9.95E-01 | 1.14 | 3.44E-01 |
| TG(49:2) [2] | 0.86 | 3.42E-01 | 1.29 | 3.84E-01 | 1.50 | 3.58E-02 |
| TG(50:0) [2] | 0.85 | 7.85E-01 | 1.22 | 3.17E-01 | 1.44 | 1.18E-01 |
| TG(50:0) [2] | 0.97 | 8.33E-01 | 1.02 | 8.61E-01 | 1.05 | 5.45E-01 |
| TG(50:1) [2] | 0.76 | 1.13E-01 | 1.74 | 3.40E-03 | 2.28 | 4.71E-05 |
| TG(50:1) [2] | 0.84 | 7.66E-01 | 0.83 | 9.88E-01 | 0.99 | 6.93E-01 |
| TG(50:2) [2] | 0.76 | 5.24E-02 | 1.54 | 1.75E-04 | 2.02 | 1.55E-06 |
| TG(50:3) [2] | 1.02 | 4.24E-01 | 2.15 | 8.99E-05 | 2.11 | 9.41E-06 |
| TG(50:3) [2] | 0.95 | 2.96E-01 | 2.00 | 1.65E-04 | 2.12 | 9.75E-06 |
| TG(50:5) [2] | 0.66 | 7.22E-01 | 1.43 | 5.22E-02 | 2.17 | 1.27E-02 |
| TG(51:1) [2] | 0.82 | 6.90E-01 | 1.16 | 8.49E-01 | 1.41 | 3.95E-01 |
| TG(51:2) [2] | 0.78 | 3.05E-01 | 1.20 | 4.15E-01 | 1.54 | 3.43E-02 |
| TG(51:3) [2] | 0.91 | 5.67E-01 | 1.14 | 7.28E-01 | 1.26 | 2.17E-01 |
| TG(51:4) [2] | 1.12 | 9.20E-01 | 1.35 | 5.61E-01 | 1.21 | 3.69E-01 |
| TG(52:0) [2] | 1.03 | 7.94E-01 | 1.15 | 3.16E-01 | 1.12 | 6.98E-01 |
| TG(52:2) [2] | 0.80 | 1.31E-01 | 1.45 | 5.90E-03 | 1.82 | 9.35E-05 |
| TG(52:3) [2] | 0.97 | 2.32E-01 | 1.09 | 4.46E-01 | 1.12 | 2.69E-02 |
| TG(52:4) [2] | 0.98 | 8.02E-01 | 1.02 | 7.36E-01 | 1.04 | 3.89E-01 |
| TG(52:5) [2] | 1.06 | 9.82E-01 | 1.23 | 1.46E-01 | 1.16 | 2.18E-01 |
| TG(52:6) [2] | 2.35 | 2.25E-01 | 2.81 | 1.63E-02 | 1.19 | 4.03E-01 |
| TG(53:2) [2] | 0.88 | 3.48E-01 | 1.00 | 9.97E-01 | 1.13 | 3.33E-01 |
| TG(53:3) [2] | 1.07 | 6.23E-01 | 1.07 | 8.59E-01 | 1.00 | 3.52E-01 |
| TG(53:4) [2] | 1.02 | 9.91E-01 | 1.00 | 8.51E-01 | 0.98 | 7.93E-01 |
| TG(53:5) [2] | 1.24 | 1.00E+00 | 1.41 | 2.89E-01 | 1.14 | 3.02E-01 |
| TG(54:1) [2] | 0.84 | 7.03E-01 | 1.05 | 9.97E-01 | 1.26 | 6.79E-01 |
| TG(54:2) [2] | 0.95 | 2.51E-01 | 1.31 | 8.13E-02 | 1.37 | 3.20E-03 |
| TG(54:3) [2] | 1.03 | 4.09E-01 | 1.42 | 2.84E-02 | 1.39 | 2.15E-03 |
| TG(54:4) [2] | 0.89 | 5.64E-01 | 1.28 | 4.48E-01 | 1.44 | 9.73E-02 |
| TG(54:5) [2] | 0.75 | 6.56E-01 | 1.26 | 4.86E-01 | 1.68 | 1.43E-01 |
| TG(54:6) [2] | 0.66 | 5.86E-01 | 1.46 | 3.43E-01 | 2.20 | 7.16E-02 |
| TG(54:6) [2] | 0.95 | 6.45E-01 | 0.67 | 9.29E-01 | 0.71 | 4.51E-01 |
| TG(54:7) [2] | 1.28 | 9.87E-01 | 1.55 | 1.14E-01 | 1.21 | 1.67E-01 |
| TG(55:5) [2] | 0.73 | 5.45E-01 | 0.81 | 5.89E-01 | 1.10 | 9.97E-01 |
| TG(56:2) [2] | 0.80 | 2.39E-01 | 1.14 | 7.79E-01 | 1.42 | 6.05E-01 |
| TG(56:3) [2] | 0.93 | 2.74E-01 | 1.25 | 9.73E-01 | 1.35 | 2.11E-01 |
| TG(56:4) [2] | 1.03 | 4.44E-01 | 0.93 | 6.59E-01 | 0.91 | 9.35E-01 |
| TG(56:5) [2] | 1.16 | 4.48E-01 | 0.84 | 9.63E-01 | 0.73 | 3.36E-01 |
| TG(56:5) [2] | 0.92 | 8.80E-01 | 0.81 | 5.14E-01 | 0.88 | 8.13E-01 |
| TG(56:6) [2] | 1.14 | 9.97E-01 | 0.70 | 5.96E-02 | 0.62 | 7.92E-02 |
| TG(56:8) [2] | 1.20 | 5.11E-01 | 0.68 | 4.80E-01 | 0.57 | 9.18E-02 |
| TG(58:6) [2] | 0.90 | 2.79E-01 | 0.61 | 4.31E-03 | 0.68 | 1.31E-01 |
| TG(58:9) [2] | 1.45 | 4.13E-01 | 0.28 | 5.98E-03 | 0.20 | 4.59E-04 |

**Supplementary Table A3.** Statistically-significantly altered metabolite levels in female NOD mice exposed to POP mixture.

| *Common metabolites in* | | | |
| --- | --- | --- | --- |
| *Low/Ctrl*  *High/Low*  *High/Ctrl* | *Low/Ctrl*  *High/Low* | *Low/Ctrl High/Ctrl* | *High/Low*  *High/Ctrl* |
| Cholesterol | PC(34:1) | Palmitic acid | Asparagine |
| 1,5-Anhydrohexitol | PC(34:3) | PC(34:2) | Aspartic acid |
| LCA | PC(32:1) | PC(32:2) | Glutamic acid |
| PC(35:3) |  | PC(33:1) | Threonine |
| PC(36:4) |  | PC(O-36:3) | 3-Hydroxybutyric acid |
|  |  |  | Arachidonic acid |
|  |  |  | Stearic acid |
|  |  |  | Gluconic acid |
|  |  |  | Glycolic acid |
|  |  |  | Palmitoleic acid |
|  |  |  | Glycerol-3-phosphate |
|  |  |  | HDCA |
|  |  |  | CE(20:3) |
|  |  |  | LPC(16:0e) |
|  |  |  | LPC(16:0p) |
|  |  |  | LPC(16:1) |
|  |  |  | LPC(18:1) |
|  |  |  | LPC(20:3) |
|  |  |  | LPC(20:4) |
|  |  |  | LPC(22:5) |
|  |  |  | LPC(22:6) |
|  |  |  | LysoPE(18:1) |
|  |  |  | PC(16:0/16:0) |
|  |  |  | PC(18:0/18:0) |
|  |  |  | PC(18:0p/18:1(9Z)) |
|  |  |  | PC(18:0p/22:6) |
|  |  |  | PC(31:0) |
|  |  |  | PC(35:1) |
|  |  |  | PC(35:2) |
|  |  |  | PC(36:1) |
|  |  |  | PC(36:2) |
|  |  |  | PC(36:3) |
|  |  |  | PC(37:1) |
|  |  |  | PC(37:2) |
|  |  |  | PC(37:3) |
|  |  |  | PC(37:4) |
|  |  |  | PC(38:2) |
|  |  |  | PC(38:3) |
|  |  |  | PC(38:4) |
|  |  |  | PC(38:5) |
|  |  |  | PC(38:6) |
|  |  |  | PC(39:6) |
|  |  |  | PC(40:5) |
|  |  |  | PC(40:6) |
|  |  |  | PC(40:7) |
|  |  |  | PC(40:8) |
|  |  |  | PC(O-32:0) |
|  |  |  | PC(O-32:1) |
|  |  |  | PC(O-34:2) |
|  |  |  | PC(O-36:4) |
|  |  |  | PC(O-36:5) |
|  |  |  | PC(O-38:4) |
|  |  |  | PC(O-38:5) |
|  |  |  | PC(O-38:6) |
|  |  |  | PC(O-40:6) |
|  |  |  | PC(P-18:0/22:6) |
|  |  |  | PE(O-38:5) or PE(P-38:4) |
|  |  |  | PE(P-18:0/22:6) |
|  |  |  | SM(d16:1/18:1) or SM(d18:2/16:0) |
|  |  |  | SM(d18:0/16:0) |
|  |  |  | SM(d18:1/24:0) |
|  |  |  | SM(d32:1) |
|  |  |  | SM(d33:1) |
|  |  |  | SM(d34:1) |
|  |  |  | SM(d36:1) |
|  |  |  | SM(d36:2) |
|  |  |  | SM(d38:2) |
|  |  |  | SM(d40:1) |
|  |  |  | SM(d41:1) |
|  |  |  | SM(d41:2) |
|  |  |  | SM(d42:2) |
|  |  |  | TG(14:0/16:0/18:1) |
|  |  |  | TG(14:0/18:1/18:1) |
|  |  |  | TG(14:0/18:2/18:2) |
|  |  |  | TG(18:1/12:0/18:1) or TG(18:2/16:0/14:0) |
|  |  |  | TG(18:1/18:1/16:0) |
|  |  |  | TG(18:1/18:1/18:1) |
|  |  |  | TG(18:1/18:1/22:6) |
|  |  |  | TG(18:2/18:2/18:2) or TG(18:3/18:2/18:1) |
|  |  |  | TG(48:1) |
|  |  |  | TG(48:3) |
|  |  |  | TG(50:1) |
|  |  |  | TG(50:2) |
|  |  |  | TG(50:3) |
|  |  |  | TG(52:2) |
|  |  |  | TG(54:3) |
|  |  |  | TG(58:9) |

- 1. **Figures**


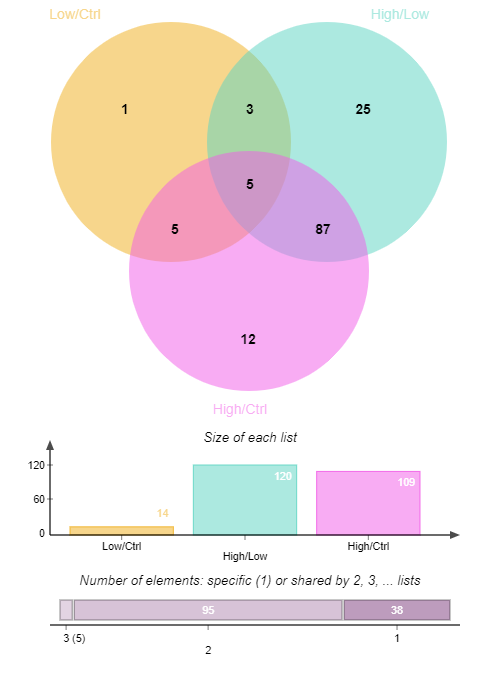


**Supplementary Figure A1.** Venn diagram showing the number of metabolites commonly dysregulated in low exposure, high exposure and control NOD mouse groups.


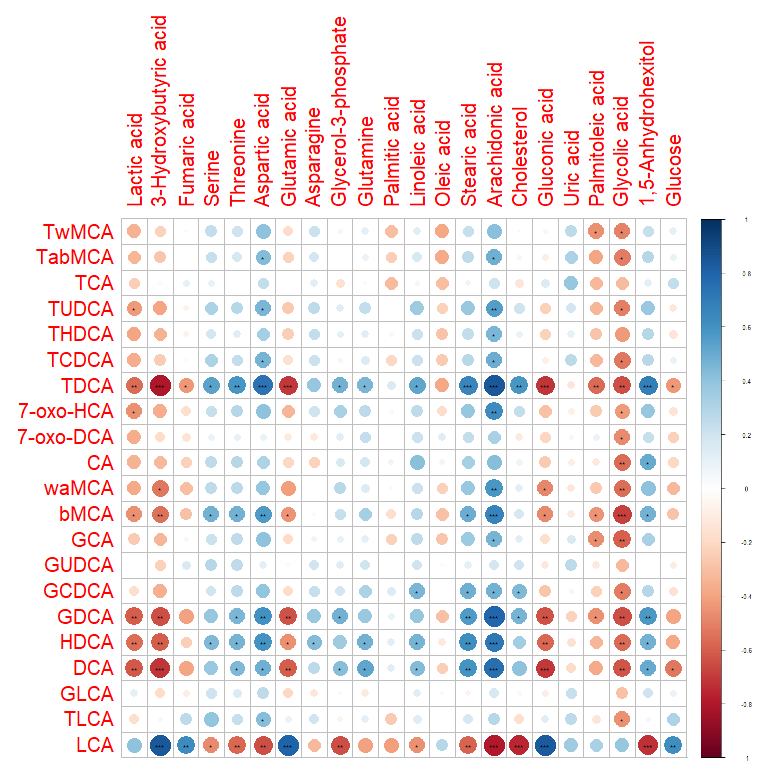


**Supplementary Figure A2.** Spearman’s correlation plot between polar metabolites and BAs. Significant correlations marked with * p<0.05, ** p<0.01 and *** p<0.001. Positive correlation showed in blue and negative in red.


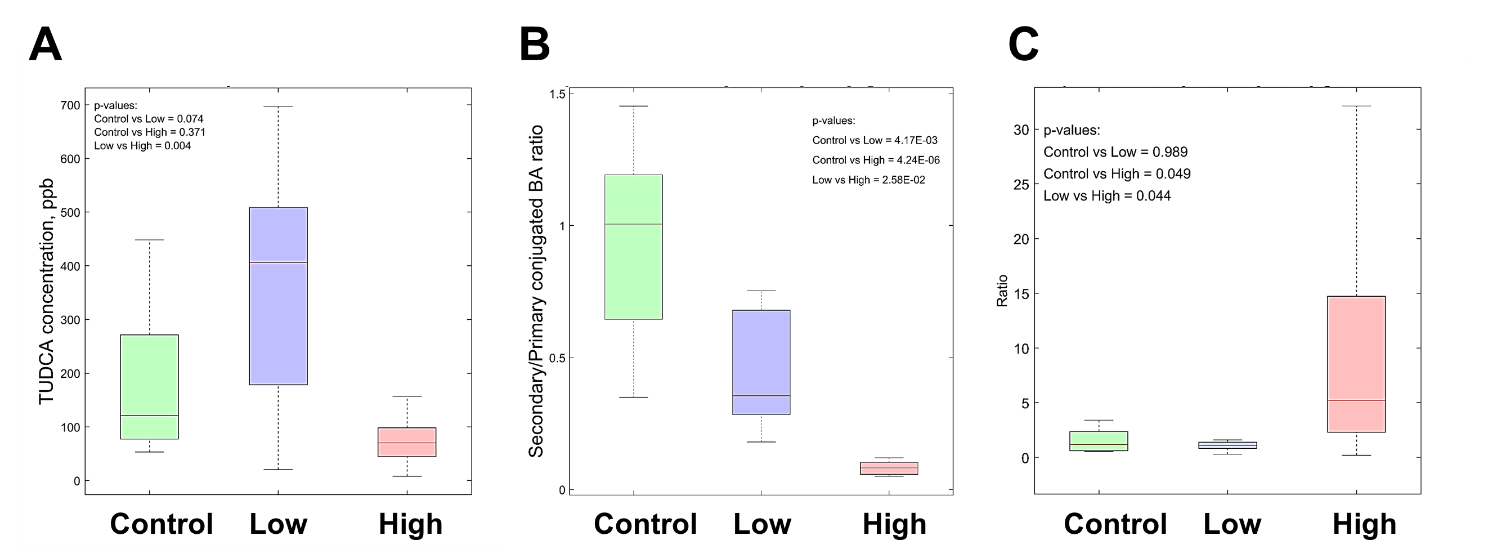


**Supplementary Figure A3.** Boxplots of A) TUDCA showing non-monotonic dysregulation in low and high exposure NOD mouse groups, B) ratio between secondary and primary conjugated BAs in control and exposed NOD mouse groups and C) ratio between secondary and primary non-conjugated BAs in control and exposed groups of NOD mice
